# From data to interpretable models: machine learning for soil moisture forecasting

**DOI:** 10.1007/s41060-022-00347-8

**Published:** 2022-08-31

**Authors:** Aniruddha Basak, Kevin M. Schmidt, Ole Jakob Mengshoel

**Affiliations:** 1 Carnegie Mellon University, Pittsburgh, USA; 2 Geology, Minerals, Energy, and Geophysics Science Center, U. S. Geological Survey, Moffett Field, California USA; 3 Department of Computer Science, Norwegian University of Science and Technology, Trondheim, Norway

**Keywords:** Soil moisture forecasting, Post-fire landslides, Data analysis, Model optimization and fitting, Monitoring, Interpretable machine learning

## Abstract

Soil moisture is critical to agricultural business, ecosystem health, and certain hydrologically driven natural disasters. Monitoring data, though, is prone to instrumental noise, wide ranging extrema, and nonstationary response to rainfall where ground conditions change. Furthermore, existing soil moisture models generally forecast poorly for time periods greater than a few hours. To improve such forecasts, we introduce two data-driven models, the Naive Accumulative Representation (NAR) and the Additive Exponential Accumulative Representation (AEAR). Both of these models are rooted in deterministic, physically based hydrology, and we study their capabilities in forecasting soil moisture over time periods longer than a few hours. Learned model parameters represent the physically based unsaturated hydrological redistribution processes of gravity and suction. We validate our models using soil moisture and rainfall time series data collected from a steep gradient, post-wildfire site in southern California. Data analysis is complicated by rapid landscape change observed in steep, burned hillslopes in response to even small to moderate rain events. The proposed NAR and AEAR models are, in forecasting experiments, shown to be competitive with several established and state-of-the-art baselines. The AEAR model fits the data well for three distinct soil textures at variable depths below the ground surface (5, 15, and 30 cm). Similar robust results are demonstrated in controlled, laboratory-based experiments. Our AEAR model includes readily interpretable hydrologic parameters and provides more accurate forecasts than existing models for time horizons of 10–24 h. Such extended periods of warning for natural disasters, such as floods and landslides, provide actionable knowledge to reduce loss of life and property.

## Introduction

Soil moisture dynamics are essential to understanding agriculture, ecology, and natural disasters such as drought, landslides, and floods [32, 51, 60]. Agricultural productivity, for instance, requires sufficient drainage to minimize soil saturation and control salinity. Managing irrigation and soil moisture directly influences crop yields. Improved soil moisture forecasting in response to rainfall enables water managers to optimize irrigation schedules, water uptake by plants, and cost.

**Fig. 1:**
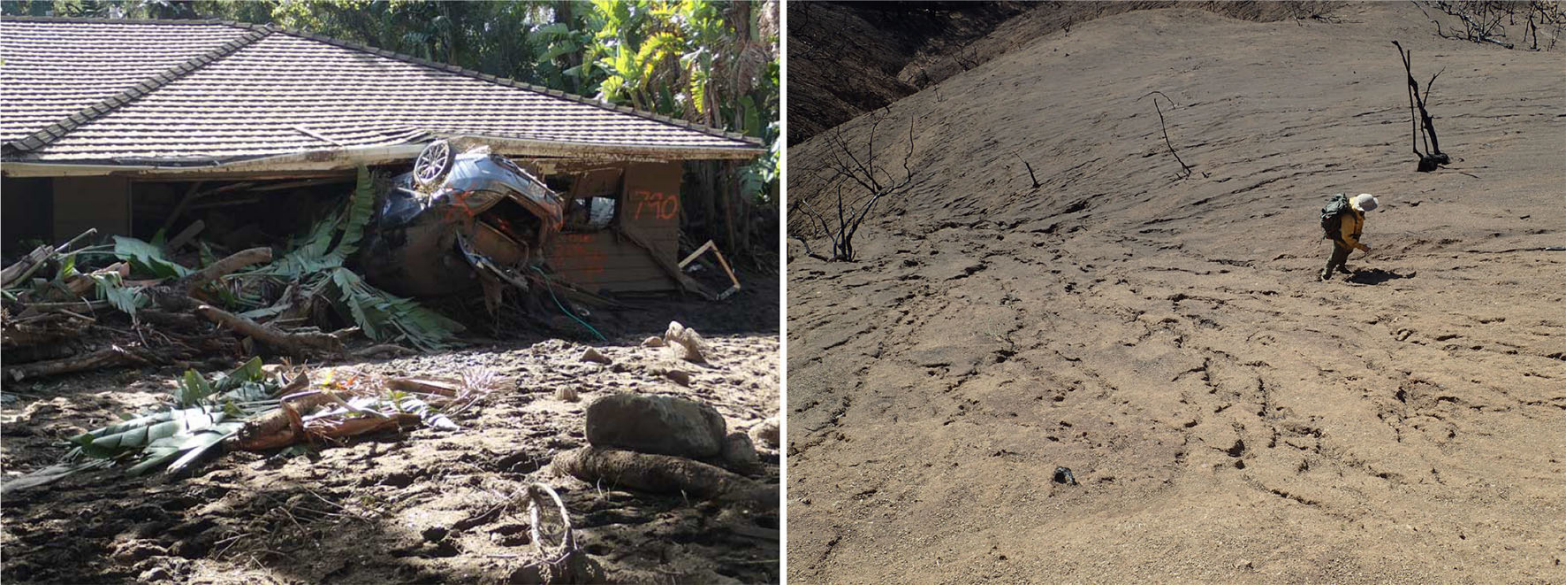
Left: Destructive post-Thomas Fire debris flow impacts of January 9, 2018 in Montecito, California. Right: U.S. Geological Survey researcher responds to deadly January 9, 2018, debris flows in Montecito, California. Distributed rainfall runoff in the absence of vegetation allows readily erodible material to transport downslope gaining mass and velocity (momentum) through the channel network resulting in damaging and deadly debris flows. Photographs by USGS

Soil moisture also moderates certain natural disasters. Post-wildfire landscapes exhibit dry soils, decreased infiltration rates, and vegetation removal by fire reducing soil-stabilizing cohesive reinforcement. These factors increase erosion and debris-flow susceptibility during subsequent rainfall [14]. Debris flows are fast-moving masses of earth materials traveling gravitationally. Because of their speed and momentum, they can be deadly and destructive (Fig. 1). Rainfall following the 2017 Thomas Fire resulted in devastating flash floods and debris flows in Montecito, California (Fig. 1) covering sections of California State Highway 101 with multiple feet of mud and debris [30]. As climate change will likely create hotter and drier conditions, future fires may result in elevated burn severities and subsequent storms may exhibit greater rainfall. In the western USA, substantial increases in post-fire risks due to extreme rainfall are predicted by the mid-twenty-first century [72].

The National Oceanic and Atmospheric Administration (NOAA) and the US Geological Survey (USGS) operate a flash flood and debris flow early warning system for recently burned areas in southern California.^1^ Leveraging the Flash Flood Monitoring and Prediction protocols of the National Weather Service (NWS), this early warning system appraises precipitation estimates with empirical intensity–duration thresholds from prior debris flow events. As evacuations occur to minimize loss of life, refining thresholds of when and where advisories are issued is imperative for hazard delineation and public safety [27].

To improve the understanding of soil moisture on earth, the National Aeronautics and Space Administration (NASA) launched a Soil Moisture Active Passive (SMAP) satellite in 2015. SMAP’s radar and radiometer remotely sense ground-surface soil moisture, creating comprehensive but coarse-scale maps. The European Space Agency (ESA) runs an Earth observation program, Copernicus, which also collects soil moisture measurements. ESA has, as of year 2022, accumulated and served over four decades of global soil moisture measurements.^2^

Soil moisture may fluctuate rapidly over time with substantial vertical and lateral variation within a soil column. Such variability can be measured by probes exploiting the contrast between dielectric properties of liquid water and dry soil to estimate volumetric water content (VWC). The literature uses three different terms: “volumetric water content (VWC),” “volumetric soil moisture,” and “soil moisture” to describe the same property. We simply adopt soil moisture. Although time series of wetting and drying in response to rainfall can be measured by dielectric probes, it remains challenging to forecast such response to incoming rain. Using observed soil moisture data from a site with changing ground conditions following fire and rainfall, this work aims to identify models capable of forecasting moisture response under non-stationary conditions over timeframes applicable to warning systems.

**Fig. 2:**
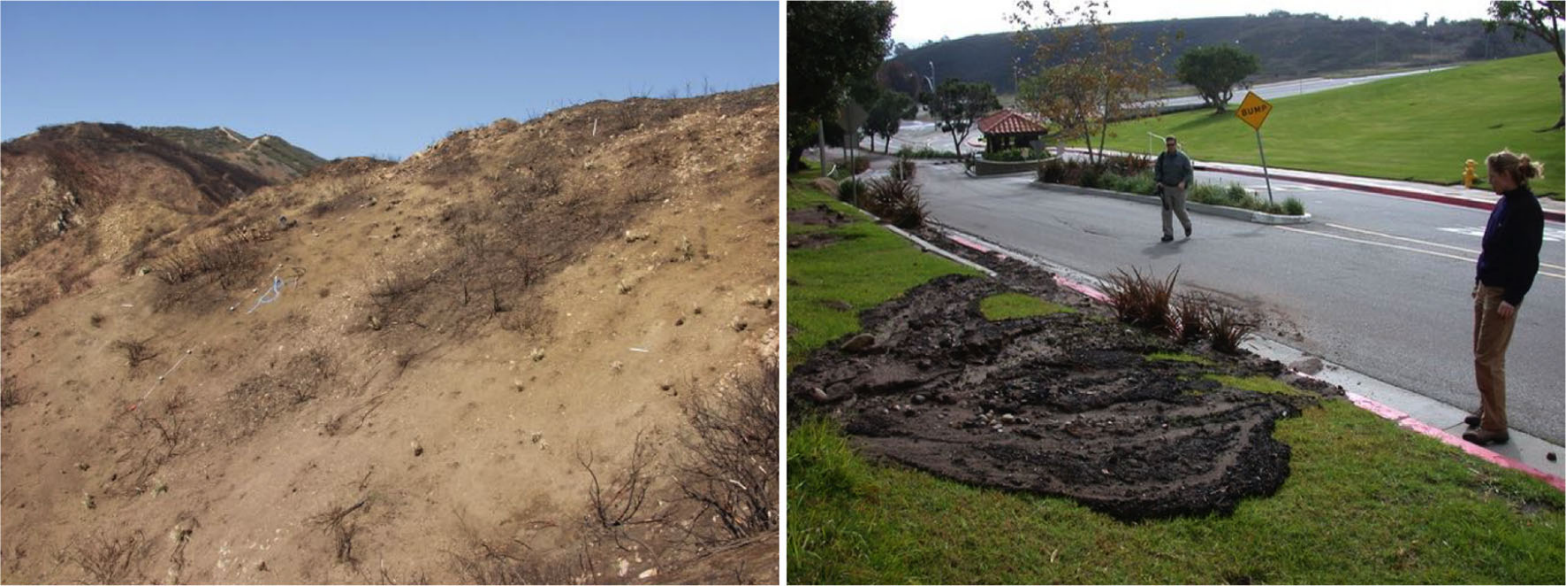
Left: Photograph of a burned hillslope with rainfall, overland flow, and soil moisture monitoring instrumentation after the 2007 Canyon fire, but before rainfall, on the Pepperdine University Malibu, California campus. Right: Photograph from December 19, 2007, of a small post-fire debris flow and flood on the Pepperdine University campus following minor rainfall measured by USGS monitoring array to left. Photographs by USGS

While theoretical models can quantify rainfall and runoff (e.g., Richards’ equation [59]), site-specific field conditions and soil moisture hysteresis with time preclude their simple use. Taking a more data-driven approach, attempts have been made to forecast soil moisture or related parameters using time-series (e.g., autoregressive integrated moving average or ARIMA [2]) or deep machine learning models (e.g., Long Short-Term Memory or LSTM [51]). Unfortunately, these models do not inherently reflect hydrological processes in an interpretable fashion [39, 49, 62]. The Antecedent Water Index (AWI) model [16, 74], though, fits soil moisture time-series data to extend beyond the period of record while providing meaningful, transparent information on hydrologic parameters. However, we establish that the existing AWI model performs poorly over time horizons exceeding a few hours. Inspired by the interpretability of hydrologic process response and ease of applicability of AWI, we present two novel AWI-based soil moisture models: the Naive Accumulative Representation (NAR) and the Additive Exponential Accumulative Representation (AEAR). The models accumulate rainfall over time to forecast soil moisture.^3^ Using these models, we apply estimation algorithms to fit diverse ranges of wetting and drying curves to extract meaningful parameters and learn about process response. AEAR and NAR model parameters are estimated from data, and the models are designed to resemble hydraulic redistribution of unsaturated soil moisture. The AEAR and NAR models are examples of domain-driven data mining and provide better hydrologic interpretability [39, 49, 62] than traditional time series models (such as ARIMA and ARMAX) and high-dimensional machine learning models (such as LSTM). Moreover, these models can be used to recursively forecast soil moisture from initial soil moisture values.

**Fig. 3:**
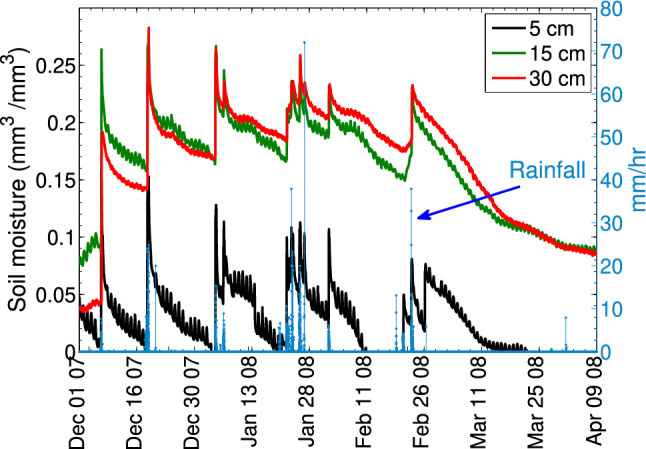
Rainfall intensity (blue line) and soil moisture measurements at three depths below ground surface (black, green, and red lines at 5, 15, and 30 cm, respectively) from the Canyon Fire field monitoring site depicted in Fig. 2

We validate our NAR and AEAR models on challenging post-fire soil moisture time-series datasets from southern California [65, 66], where rapid sediment erosion and deposition were observed following wildfire (Fig. 2). Figure 3 depicts the raw soil moisture and rainfall measurements from the monitoring array depicted in Fig. 2. The second soil moisture peak, with the highest values recorded during the monitoring period, coincides with the debris flow producing event depicted in (Fig. 2). Such rapid increases in rainfall-induced soil moisture likely reflect fast preferential water flow processes. As water enters desiccation cracks of initially dry soil, for example, it quickly delivers moisture to deeper levels. We also evaluate model performance using soil moisture data obtained from controlled laboratory experiments. Overall, the most robust forecasts were obtained with the novel AEAR model, which is both data driven and readily interpreted by earth scientists.^4^

Our main contributions are as follows:

1. Derivation of two novel accumulative rainfall soil moisture models NAR and AEAR.
2. Simulation of post-fire soil moisture response with a controlled experiment.
3. Validation of the proposed soil moisture models in field and controlled experimental datasets.
4. Comparison with benchmark time series and machine learning forecasting methods including autoregressive models and LSTM.

This paper presents complete data solutions, from real-world data collection to forecasting, that aid earth scientists in socially relevant decision making. We address the socioeconomic challenges of forecasting extreme weather events and natural disasters (e.g., Fig. 1), agricultural productivity, and climate-influenced trends that arise from soil water conditions. These problems often involve data analysis to derive critical actionable insights in the context of decision making [62], such as early warning for landslides (e.g., Fig. 2), irrigation, and infrastructure management. The topic of forecasting soil moisture is well suited to domain-driven data mining methodologies [39, 49, 62] for determining actionable knowledge for public safety and loss reduction.

The rest of the article is structured as follows: Sect. 2 presents notation, terminology, and requirements. The data analysis process and data types are discussed in Sect. 3. Section 4 introduces two novel soil moisture models, NAR and AEAR. Section 5 presents both quantitative and qualitative analysis results, including validation of model interpretability by earth scientists. We discuss related work in earth science, time series modeling and forecasting, and machine learning in Sect. 6. We conclude and outline future research opportunities in Sect. 7.

## Goals and requirements

Our primary goal is to develop readily explainable physically based models that capture soil moisture response to rainfall. These models should reflect the timing and magnitude of hydraulic redistribution from simplified gravitational and matric suction processes. After introducing notation and terminology, we discuss three model requirements.

### Notation and terminology

We consider a time series or sequence of records, $(r_0,r_1,$ $\ldots ,r_i,\ldots )$, where each record consists of a time stamp *t* and a measurement value *v*: $r = (t, v)$ or for simplicity $v_t$. For a particular dataset, we consider the following sequences:

- A sequence (or time series) of raw soil moisture data (or measurements) ${\mathscr {D}} = (D_0, \ldots ,D_i, \ldots ).$
- A sequence of processed (*i.e.,* smoothed and sub-sampled) soil moisture measurements: ${\mathscr {M}} {=} (M_0,\ldots ,M_i,\ldots )$.
- A sequence of soil moisture forecasts: $\hat{{\mathscr {M}}} = ({\hat{M}}_0,\ldots ,{\hat{M}}_i,$ $\ldots )$.
- A sequence of rainfall measurements: ${\mathscr {I}} = (I_{0},\ldots ,I_{i},\ldots )$.
- A sequence of rainfall forecasts: $\hat{{\mathscr {I}}} = ({\hat{I}}_{t^{*}},\ldots ,{\hat{I}}_{t^{*} + i},\ldots )$.

Let $t^{*}\ge 0$ and $\tau > 0$. For a point forecast for time $t^{*}$, a model should forecast soil moisture ${\hat{M}}_{t^{*} + \tau }$, given a prediction horizon $\tau$. For a sequence forecast, we typically use an *M*-value at a particular time $t^{*}$, $M_{t^{*}}$, to forecast a subsequence of $\hat{{\mathscr {M}}}$ for a time horizon of length $\tau$; thus:

1 $$\begin{aligned} {\hat{\mathscr {M}}}_{t^{*} + 1 : t^{*}+\tau } = \left( {\hat{M}}_{t^{*} + 1}, \ldots , {\hat{M}}_{t^{*}+\tau }\right) . \end{aligned}$$

We often say just forecast in our discussion since it is usually clear from the context whether a point forecast ${\hat{M}}_{t^{*} + \tau }$ or a sequence forecast ${\hat{\mathscr {M}}}_{t^{*} + 1 : t^{*}+\tau }$ is intended. Further detail is found in Sect. 4.4.

We assume that future rainfall data are available through separate forecasts, such as issued by the NWS.^5^ We focus on hillslope soil moisture response to rainfall, not on rainfall forecasting, as several well-established rainfall forecasting methods exist [61]. We incorporate no uncertainty estimates of rainfall forecasts into our models.

### Three model requirements

Models that forecast soil moisture should meet the following three criteria:

- *Interpretability*: Models need to be easily interpreted by earth scientists by having parameters based on physical processes and soil or hydrological properties. Interpretable and trustworthy models are gaining importance in artificial intelligence. By learning interpretable models, one can support high-stakes decision making for human health and safety [39, 62] and potentially avoid situations that poorly generalize beyond the test-set. Thus, we call for model-based interpretability [49, 62] when forecasting soil moisture from rainfall.
- *Data-driven*: Model parameters must be computed from data collected by current sensor technology. Both regular and irregular measurements should be supported. A model with parameters that cannot be easily estimated from, and validated against, data is less useful.
- *Accurate actionable forecasts*: A model must provide forecasts that are accurate in the medium timeframe $\tau$, which we here define with a time horizon of 5 $\le \tau \le$ 24 h, aligned with the timeframe of NWS weather forecasts. Humans can prophylactically act on the medium time scale to prepare for future events. For example, if an area is threatened by debris flows, evacuation warnings can be heeded to maximize safety.

All of the above three requirements need to be met; it is not sufficient to satisfy just two. Machine learning models, for example, are obviously data driven and may provide accurate medium-term forecasts. However, some of them are not based upon meaningful physics-based parameters interpretable by scientists. Although it may be possible to train a deep neural network from soil moisture data and apply it to compute accurate medium-term forecasts, the resulting large number of parameters precludes meaningful physical interpretations. Another model type with limited utility is one that is scientifically meaningful and data-driven, but only produces accurate short-term forecasts. The Antecedent Water Index (AWI) has been applied to such shorter duration time horizons, see details in Sects. 5 and 6.1.

Among the three requirements, there is no stipulation that models must be of a certain mathematical form. Models need not be linear, convex, or differentiable functions. Thus, we do not restrict mathematical forms nor parameter optimization methods (see Sects. 4 and 5).

## Data analysis process

**Fig. 4:**
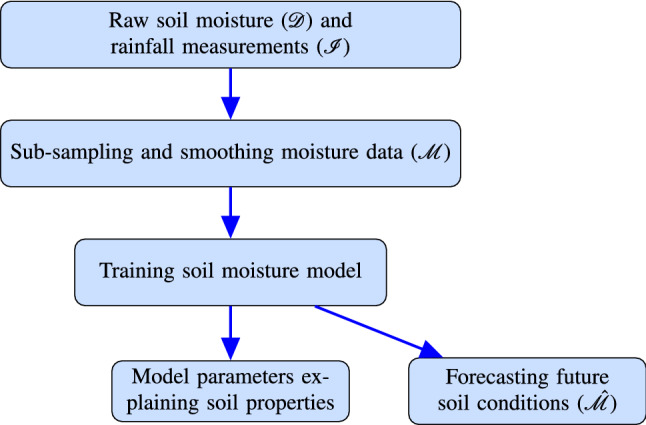
Inputs, outputs, and steps of our soil moisture data analysis process; see Sect. 3 for further explanations

In a wide range of geomorphic, hydrologic, and ecosystem contexts, a need exists for a clear data science process when characterizing soil moisture response. Figure 4 illustrates the steps of our data analysis process, from raw soil moisture measurements, via soil moisture models, to model forecasts.

- Soil moisture and rainfall measurements: These inputs are enabled by recent technology improvements, which increased the availability of affordable sensors and data loggers that aid measurement and recording of raw soil moisture data; a time series: ${\mathscr {D}} = \left( D_0,\ldots ,D_i,\ldots \right)$.
- Sub-sampling and smoothing moisture data: The preprocessing step of taking ${\mathscr {D}}$ as input (i) reduces computational demands via sub-sampling and (ii) diminishes temperature-induced and other variations from soil moisture data while preserving local maxima, or “peaks,” and local minima, or “valleys.” The result is a preprocessed time series of soil moisture measurements: ${\mathscr {M}} = \left( M_0,\ldots ,M_i,\ldots \right)$.
- Training soil moisture model: This step learns or estimates, from data ${\mathscr {M}}$, a *hydrologic model*. This model, for example AEAR, expresses soil moisture as a function of rainfall and time.
- Model parameters explaining soil properties: Using the *hydrologic model*, this step aims to validate whether model parameters provide physically interpretable information.
- Forecasting future soil conditions: Using the *hydrologic model* with rainfall forecasts and current soil water content, this step aims to accurately forecast a soil moisture sequence: $\hat{{\mathscr {M}}} = \left( {\hat{M}}_0,\ldots ,{\hat{M}}_i,\ldots \right)$.

In the remainder of Sect. 3, we discuss process inputs, outputs, and steps as summarized above and in Fig. 4.

### Raw soil moisture and rainfall measurements

We studied raw soil moisture (${\mathscr {D}}$) and rainfall measurements (${\mathscr {I}}$) at one post-fire field setting and one controlled experimental setting. We did not evaluate the temporal variability, non-stationary behavior, or entropy of rainfall at the fine scale [13, 44] in the context of post-fire erosion. Rainfall, though, is a multi-scale event with non-stationary temporal behavior that is outside the scope of this work. Similarly, we do not incorporate the variability in spatial structure of rainfall over a landscape [27]. We now present these two settings and their corresponding raw soil moisture datasets as summarized in Table 1.

**Table 1:** Summary of the six soil moisture datasets used in our analyses; see Sect. 3.1 for additional information

Location	Depth (cm)	Dataset Id
Field: Canyon Fire	5	Canyon-5
	15	Canyon-15
	30	Canyon-30
Controlled experiment: Bucket	10	Bucket-10
	20	Bucket-20
	28	Bucket-28

#### Datasets from the field (2007 Canyon Fire data)

The field monitoring dataset is composed of raw soil moisture (${\mathscr {D}}$) and rainfall (${\mathscr {I}}$) measurements from the Santa Monica mountains near the town of Malibu and Pepperdine University in southern California [19, 64].^6^ These datasets are published as a USGS Data Release [66]. The study site was within the 2007 Canyon Fire that burned over 4500 acres and destroyed 22 structures. Prior to the fire, the area was covered by chaparral vegetation. However, the fire disturbance removed almost all of the vegetation and changed soil infiltration properties. Hillslopes within the study site are steep with gradients up to 0.9. The colluvial soils are generally less than 0.5 m thick, overlying sedimentary rock. These soils have much higher infiltration rates than the underlying Miocene bedrock. These underlying sediments produced a range of colluvial soil types with a grain size of 40–60% sand, classified as loams to sandy loams. Due to the steep landscape with high infiltration rates characteristic of sandy soils, rapid soil moisture response is observed following rain events. These factors promote rapid wetting with slow drying of soil from hydraulic redistribution following rain.

Post-fire hydrologic response is more complicated than equivalent response of unburned settings, arising from fire-induced changes to the soil and vegetation. Such complicated soil moisture response to rainfall, though, ensures that the resulting models developed are resilient and flexible for application to a variety of field settings.

Soil moisture was field-measured using probes (from Decagon Devices Inc.^7^) connected to data loggers. Probes measured volumetric water content (VWC), a measure of ratio of water volume to soil volume, by estimating the dielectric constant of the media using capacitance and frequency domain technology [35]. Probes were placed at three depths below the ground surface (5, 15, and 30 cm), to represent different soil horizons of varying texture. Soil moisture was recorded every 2 min on nearby data loggers. Tipping bucket rain gauges, also connected to data loggers, provided precipitation data on an irregular schedule recorded in response to rainfall. Soil moisture and rainfall data from December 2007 to April 2008 are shown in Fig. 3 [66] illustrating several major and minor precipitation events along with the corresponding wetting and drying of soil.

#### Datasets from controlled experiment (bucket data)

To study soil moisture dynamics in a more controlled setting, experiments were performed at the Carnegie Mellon University campus in the NASA Research Park, near Mountain View, California. We filled a 5 gallon plastic bucket with sand (grain size $<1$ mm) and gravel (grain size $\approx$ 25–35 mm) in proportions to mimic the hillslope sediment present at the 2007 Canyon Fire study site. To approximate the post-fire scenario, we started this controlled experiment with very dry material. Three VWC sensors placed at 10, 20, and 28 cm depths recorded raw soil moisture ${\mathscr {D}}$. The VWC sensors were identical to the Decagon probes used in the field experiment, see Sect. 3.1.1. The experiment was conducted outdoors exposed to normal weather conditions.

Two types of data records make up the “rainfall” sequence ${\mathscr {I}}$. First, we manually added measured amounts of water in intervals bringing soil to near-saturated conditions. The manual process was executed to simulate the rainfall observed in Fig. 3. Second, some natural rainfall events during the time of the experiment also added water to the bucket. We quantified natural rainfall intensities and cumulative totals from Weather Underground^8^ historical data. For the controlled experiment, there was no vegetation in the soil and the rainfall events were moderate. Overall, soil moisture changes were less abrupt compared to the Canyon Fire dataset, likely resulting from the presence of an impervious container with hydrologic redistribution limited to evaporation from top of the bucket and no plant water uptake.

### Preprocessing of soil moisture datasets

The purpose of preprocessing raw data ${\mathscr {D}}$ to processed ${\mathscr {M}}$ is twofold:

- to smooth out diurnal variations, sensor noise, and sensor failure while preserving peaks and valleys; and
- to achieve computational efficiency, supporting rapid and interactive data analysis.

To achieve these goals, two preprocessing steps, namely *sub-sampling* and *smoothing*, are implemented as follows.

**Fig. 5:**
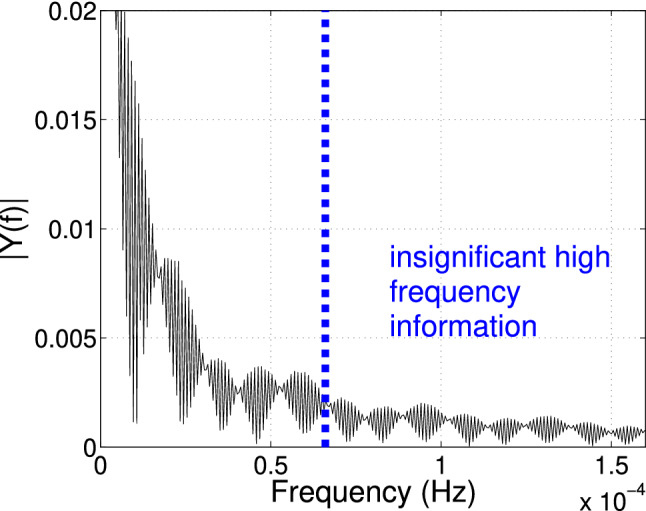
Single-sided amplitude spectrum of 5 cm soil moisture data from the Canyon Fire study area

First, *sub-sampling* to 20-min intervals reduces the analytical computational cost. The 2 min sampling rate exceeds our analytical needs. We determined that the 20 min subsampled rate was sufficient for fast completion of all computational analysis without losing significant signal. We estimated the maximum sub-sampling rate using the Nyquist–Shannon sampling theorem [50]. Figure 5 shows the amplitude spectrum of the 5 cm soil moisture data, collected from Canyon Fire field monitoring. Diurnal soil moisture variations are highest near the ground surface due to direct exposure to sunlight and wind-driven evaporation. Therefore, the probe closest to the ground surface imposes the strongest constraint for subsampling with minimal loss of information. Most of the power of the moisture signal was limited to 0.6e−4 Hz (time period = 4.6 h). Hence, according to the Nyquist criterion, proper reconstruction of the signal requires one sample every 9.2 hour. In our experiments, the sub-sampling amounted to conservatively retaining 1 record per 20 min, sufficient for fast completion of all analytical steps.

Second, *smoothing* reduces the diurnal and other variations while retaining important signals, i.e., the peaks (local maxima) and valleys (local minima). Raw, environmental monitoring data, such as near-surface VWC measurements in shallow soils determined with dielectric sensors, are prone to temperature-induced fluctuations. These diurnal variations add complexity in estimating moisture response functions. Reduction in temperature-induced noise from measurements using traditional smoothing methods alters the moisture response peaks which convey critical limiting conditions of soil [4]. For predicting landslides and runoff-driven erosion, peak values provide critical information. On the other hand, smoothed data make model fitting easier since the high-frequency diurnal variations as well as other sensor noise are suppressed. We experimented with various traditional smoothing techniques such as moving averages, spline, and LOESS smoothing. When applied to our challenging soil moisture datasets, we found that all gave similar results: diminished peaks and lost details [4]. Thus, we extended STL, seasonal decomposition using local regression [9], to deconstruct a soil moisture dataset. Building on STL, we developed the HyperSTL extrema-preserving smoothing technique [4, 36] to smooth soil moisture time series without significantly distorting or diminishing the extreme values (peaks and valleys) in the soil moisture time series.

### Training soil moisture model

Using the sub-sampled smooth data, we train *hydrologic models* on moisture data irrespective of soil horizon depth. A detailed description of the NAR and AEAR models is provided in Sect. 4. For model fitting, we split a sub-sampled and smoothed soil moisture sequence ${\mathscr {M}}$ into a training sequence $\mathscr {M}_\mathscr {T}$ and a prediction (or test) sequence $\mathscr {M}_\mathscr {P}$ as follows: $\mathscr {M}_\mathscr {T} = \left( {M}_0,\ldots ,{M}_{i}\right)$ and $\mathscr {M}_\mathscr {P} = \left( {M}_{i+1},\ldots ,{M}_{k}\right)$. The wetting and drying of soil are driven by rainfall events. We therefore split the data into $\mathscr {M}_\mathscr {T}$ and $\mathscr {M}_\mathscr {P}$ as per major rainfall events. We keep the first two wetting and drying cycles in $\mathscr {M}_\mathscr {T}$, and the rest in $\mathscr {M}_\mathscr {P}$. We assume that accurate rainfall data ${\mathscr {I}}$ are available throughout.

We train a model by optimizing its parameters and then forecasting. The Differential Evolution (DE) optimization method was selected after benchmarking several methods in pilot experiments (see Sect. 5.2) to solve problems involved with model fitting. For example, to estimate parameters of the AEAR model (6), AEAR optimization (10) based on the training sequences $\mathscr {M}_\mathscr {T}$ and $\mathscr {I}_\mathscr {T}$ is performed first.

It is important to note that we need two different procedures to train the accumulative rainfall models from regular and irregular training data, respectively. We show the distinction in the pseudocode presented in Algorithm 1 and discuss further in Sect. 4.4. When regular data are available, the accumulative model can use the true observation history to make a future prediction. However, in case of irregular data, the model needs to reuse the predicted values of the recent past to estimate a future soil moisture value.
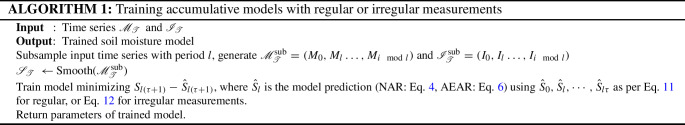


### Forecasting soil moisture conditions

Depending on the local soil characteristics and infiltration rates, a range of soil moisture conditions can initiate highly mobile, fast moving debris flows (see Figs. 1, 2). Hence, forecasting soil moisture is crucial to estimating the timing of such landslides.

Using a trained soil moisture model (*e.g.,* NAR or AEAR), forecasts ${\hat{\mathscr {M}}_P}$ for the sequence $\mathscr {M}_\mathscr {P}$ are computed and evaluated. The nature of the evaluations varies and ranges from visual comparisons to evaluating forecasting errors (standard error and maximum absolute error). Our empirical results in Sect. 5 show multiple forecasting results under both the *regular* and *irregular* settings.

### Interpreting soil moisture model parameters

As one of our requirements is for interpretability, both the NAR and AEAR models incorporate parameters representative of unsaturated soil hydrologic processes. Consequently, the trained model parameters explain processes of water infiltration and moisture redistribution in soil. Such processes control increases and decreases in soil moisture by gradients in suction and gravity. Section 5.8 discusses connections between model parameters and soil moisture.

## Soil moisture models

The NAR and AEAR models were developed by a multi-disciplinary team consisting of earth and data scientists. The goals of these models are to be interpretable (physics-based), be data driven, and produce accurate predictions (see requirements in Sect. 2). The model parameters, discussed below, are estimated from data as reported in Sect. 5. Although intended to reflect physical hydrologic process response, the models do not explicitly account for soil preferential flow paths, macropores, nor other features driving instabilities in hydrological response. Rather they accumulate rainfall over a time interval with drainage coefficients.

Before embarking on a presentation of the NAR and AEAR models in Sects. 4.2 and 4.3, respectively, we discuss SEM and its optimization in Sect. 4.1.

### Optimizing the simple exponential model (SEM)

It is important to understand the pros and cons of the simple exponential model (SEM), as it forms the foundation for the NAR and AEAR models. Wilson and Wieczorek [74] developed SEM, or an antecedent water index (AWI), based on an analogy of water flow through a leaky bucket:

2 $$\begin{aligned} M^S_{t}&= M^S_{t-1} e^{-k_d \Delta t} + \frac{I_t}{k_d}(1- e^{-k_d \Delta t} ), \end{aligned}$$

where the superscript *S* indicates SEM. The model is a simple sum of two exponentials where the first term in (2) represents recession of AWI after rain ceases and the second term represents increase in AWI due to additional rainfall. An instantaneous rainfall measurement at time *t* is denoted by $I_t$. The drainage coefficient, $k_d$, is a single exponential parameter present in both terms of Eq. (2).

We fit the SEM model in (2) to the training data by solving a mean squared error minimization problem:

3 $$\begin{aligned} {\hat{k}}_d = \min _{k_d} \sum _{t=1}^T ({M}_t - M^S_t(k_d; M_{t-1}, I_t))^2. \end{aligned}$$

Here, the *M*- and *I*-values are in the data (${\mathscr {M}}, {\mathscr {I}}$), and we optimize to find ${\hat{k}}_d$ corresponding to $k_d$ in (2). We solve this optimization problem via stochastic methods, see Sect. 5.2.

The SEM model has certain limitations. While it predicts near-surface soil moisture with reasonable accuracy, it often fails to explain complex behavior of soil moisture in deeper layers. The SEM model (2) has just one parameter related to soil moisture response, $k_d$. In other words, the SEM model has the same drainage coefficient $k_d$ for both increase and decrease in soil moisture. This significant assumption regarding $k_d$ is inconsistent with hydrological theory, as well as with field and laboratory observations.

Specifically, when fitting models to soil moisture data, the SEM model (2) forecasts near-surface soil moisture with reasonable accuracy but fails to forecast responses in longer hourly to daily timeframes, nor does it represent in an interpretable manner the more complex behavior of soil moisture in deeper layers. Typically, the soil moisture variations in deeper soil ($\ge 15$ cm) do not resemble an exponential curve as used in (2). Experimental results providing further details are in Sect. 5, for example, in Sect. 5.5. To address these limitations, we now introduce our models in Sects. 4.2 and 4.3.

### Novel model: Naive Accumulative Representation (NAR)

We expand upon the established SEM model by introducing a temporal forecast horizon and a wetting rate to accompany the drainage coefficient. Rainfall measured by commonly used tipping bucket gauges generates time series measurements separated by null values. These null values represent rainfall hiatuses during otherwise continuous events. Thus, instantaneous rainfall values in (2) create artificial “bumps” in soil moisture response. To address this issue, we accumulate rainfall over a temporal forecast horizon $\tau$.

#### Definition 1

The Naive Accumulative Representation (**NAR**) model is:

4 $$\begin{aligned} M^N_{t} = M^N_{t-\tau } e^{-k_d \tau } + \sum _{j = 0}^\tau \left[ \frac{I_{t-j}}{\eta }(1- e^{-k_w j})e^{-k_d j} \right] , \end{aligned}$$

where $k_d$ is the drying rate and $k_w$ the wetting rate. Thus, we have two constants $k_d$ and $k_w$ in contrast to just one constant in the original SEM model (2). Rainfall is measured in inches per hour, and soil moisture is a unitless quantity representing the volume of water within a volume of soil. Since rainfall and soil moisture respond at different time scales, we introduce $\eta$ as an unknown proportionality constant in (4). Although all rainfall measurements in the prediction horizon $\tau$ contribute to an increase in soil moisture, simultaneous evapotranspiration and hydraulic redistribution mechanisms in soil moderate the effect of recent rainfall. Thus, we add a drying factor $e^{-k_d j}$ in the second term of Eq. 4.

To fit the novel **NAR** model to data according to (12), we use the following optimization formulation.

5 $$\begin{aligned} ({\hat{k}}_d, {\hat{k}}_w,{\hat{\eta }})&= \min _{k_d, k_w, \eta } \sum _{t=1}^T ({M}_t \nonumber \\&\quad - M^N_t(k_d, k_w, \eta ; M^N_{t-\tau }, I_t,\cdots ,I_{t-\tau }))^2. \end{aligned}$$

Again, the *M*- and *I*-values are in the data, and we optimize to learn parameters ${\hat{k}}_d$, ${\hat{k}}_w$, and ${\hat{\eta }}$. The objective function in (5) is non-convex, and therefore, we employ stochastic optimization methods to solve the optimization problem (see Sect. 5.2).

While reasonable, the NAR model has some limitations (see empirical results in Sect. 5). Specifically, while the model can fit moisture variations in a shallow soil layer (5 cm depth), it performs poorly for the deeper layers (15 and 30 cm depths), which are shielded from evaporation and have generally finer grained soil textures. The NAR model is a stepping stone for the AEAR model, discussed next.

### Novel model: Additive Exponential Accumulative Representation (AEAR)

We now introduce a sum of exponential functions model, AEAR, to forecast a wider range of soil moisture conditions compared to the SEM and NAR models. To obtain the AEAR model in (6), we substitute the single exponential in the first term of the NAR model in (5) by a weighted sum of two exponentials. Experiments with more than two exponentials in the weighted sum showed that there is no significant performance gain with the added complexity.

#### Definition 2

The Additive Exponential Accumulative Rainfall (**AEAR**) model is:

6 $$\begin{aligned} M^A_{t}&= M^A_{t-\tau } \bigg [\alpha e^{-k_{s} \tau } + (1-\alpha ) e^{-k_{g} \tau } \bigg ] \nonumber \\&\quad +\sum _{j = 0}^\tau \left[ \frac{I_{t-j}}{\eta }(1- e^{-k_w j})e^{-k_{s} j} \right] . \end{aligned}$$

The first exponential in (6), with drainage coefficient $k_s$, represents the steep redistribution decay from the combination of strong suction gradients between wet and dry soil and gravitationally driven moisture redistribution. The second exponential in (6), with $k_g$, accounts for the gradual (slower) redistribution decay from low suction gradients, with unsaturated moisture movement dominated by gravity. The relative weighting of these two terms is controlled by a time-varying weight $\alpha$ defined by:

7 $$\begin{aligned}&\alpha = \sum _{j = 0}^\tau \left[ \frac{I_{t-j}}{\eta }(1- e^{-k_w j})e^{-k_{g} j} \right] . \end{aligned}$$

With the introduction of rainfall derived water following dry periods, suction gradients from capillary potential are strong at wetting fronts as newly added water moves towards drier soil [59]. High rates of change in soil moisture accompany such wetting fronts. During long absences of rain, such suction gradients, though, weaken as moisture is redistributed. Thus, $\alpha$ needs to be proportional to the cumulative rainfall amount. To make the variation in $\alpha$ smoother over time, we use the accumulative rainfall term ($\sum _{j = 0}^\tau I_{t-j} f(j)$, where *f*(*j*) captures the remaining multiplier) in (7) instead of instantaneous rainfall values.

There is, in fact, a resemblance between the ARMAX model and the AEAR model (6). A standard form of ARMAX model is the following:

8 $$\begin{aligned} M_t = \varepsilon _t + \sum _{i=1}^p \varphi _i M_{t-i} + \sum _{i=1}^q \theta _i \varepsilon _{t-i} + \sum _{i=1}^b \eta _i I_{t-i}. \end{aligned}$$

An interesting relationship exists between the AEAR and ARMAX models, illustrated by the following results.

#### Theorem 1

The AEAR model (Eq. 6) reduces to an ARMAX model if $\alpha$ is time invariant.

#### Proof

Equation 6 can be written as

9 $$\begin{aligned} M^A_{t} =&\sum _{i = 1}^\tau \phi _i M^A_{t-i} + \sum _{j=0}^\tau \gamma _j I_{t-j}, \end{aligned}$$

where $\phi _1 = \phi _2 = \cdots = \phi _{t-\tau +1} = 0$ and $\phi _{t-\tau } = [\alpha e^{-k_{s} \tau } + (1-\alpha ) e^{-k_{g} \tau } ]$ are the parameters of the ARMAX autoregressive terms. Here rainfall corresponds to the exogenous input, and the parameters are $\gamma _j = (1- e^{-k_w j})e^{-k_{s} j}/\eta$. If $\alpha$ is time invariant, $\phi _{t-\tau }$ reduces to an unknown constant. In this case, Eq. 9 is identical to the ARMAX model. $\square$

Unfortunately, the reformulation of the AEAR model to an ARMAX model results in the absorption of the AEAR parameters $k_s$ and $k_g$ into one time-invariant ARMAX term $\phi _{t-\tau }$. Once these parameters are aggregated, the model will lose resemblance to the hydrological processes, where $k_s$ and $k_g$ have clear physical meanings as discussed above. Adhering to our interpretability requirement (see Sect. 2.2), we thus favor the AEAR model over ARMAX.

The AEAR model was fit to the data by using mean squared error minimization:

10 $$\begin{aligned} ({\hat{k}}_s, {\hat{k}}_g, {\hat{k}}_w,{\hat{\eta }})&= \min _{k_d, k_w, \eta } \sum _{t=1}^T ({M}_t \nonumber \\&\quad - M^A_t(k_s, k_g, k_w, \eta ; M^N_{t-\tau }, I_t,\cdots ,I_{t-\tau }))^2, \nonumber \\&\quad \text {subject to } k_s > k_g. \end{aligned}$$

The *M*- and *I*-values are in the data as before, and we estimate the parameters ${\hat{k}}_s$, ${\hat{k}}_g$, ${\hat{k}}_w$, and ${\hat{\eta }}$. We impose the constraint $k_s > k_g$ to reflect the hydrological phenomenon that a suction gradient $k_s$ is stronger than the gradual, gravity dominated redistribution $k_g$. With no convexity guarantees, we use stochastic optimization methods (see Sect. 5.2) to solve the minimization problem in (10).

**Fig. 6:**
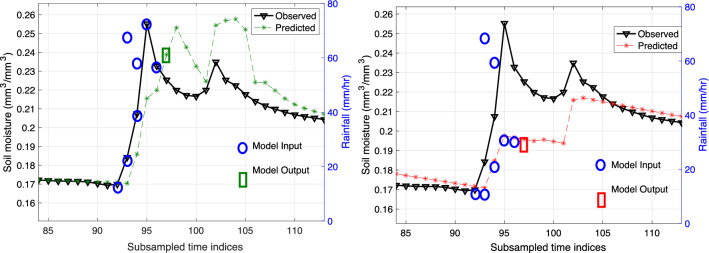
Two different forecasts at $t^{*} + \tau$, namely ${M}_{t^{*} + \tau }$ = ${M}_{97}$, illustrating the difference between the regular and irregular approaches. For clarity, we are showing regular observed measurements (model inputs, blue circles) for both cases, even though they are only present for forecasting purposes in the regular case. Left: Forecast for ${M}_{97}$ (model output, green rectangle) using regular measurement ${M}_{t^{*}}$ = ${M}_{94}$ $\approx$ 0.213. Right: Forecast for ${M}_{97}$ (model output, red rectangle) using an irregular “pseudo-measurement” ${\hat{M}}_{t^{*}}$ = ${\hat{M}}_{94}$ $\approx$ 0.185

### Forecasting approaches

For a forecast taking place at time $t^{*}$, given a prediction horizon $\tau$, a model should forecast soil moisture ${\hat{M}}_{t^{*} + \tau }$. In our case, $\tau$ is on the order of hours. Forecasts can be performed using at least two approaches, and it is desired that both are supported. There is at time $t^{*}$ either (i) an actual soil moisture measurement ${M}_{t^{*}}$ or (ii) a predicted soil moisture value ${\hat{M}}_{t^{*}}$. In addition, there are rainfall measurements in ${\mathscr {I}}$ or forecasts in $\hat{{\mathscr {I}}}$ up to time $t^{*} + \tau$. The following two formal definitions reflect (i) and (ii), respectively.

#### Definition 3

If *regular* (or frequent) raw soil moisture measurements are available, observations ${{\mathscr {D}}}$ can be processed to predict ${\hat{M}}_{t^{*} + \tau }$ using ${M}_{t^{*}}$ via a model *f*:

11 $$\begin{aligned} {\hat{M}}_{t^{*} + \tau } = f({M}_{t^{*}}, I_{t^{*}},{\hat{I}}_{t^{*} + 1}, \ldots , {\hat{I}}_{t^{*} + \tau }), \quad \forall t^{*} > 0. \end{aligned}$$

If measurements are sampled regularly or “continuously” stored in a data logger or telemetered, (11) can be used.

In the case of irregular measurements, the measurement ${M}_{t^{*}}$ of (11) is missing (else we have the regular case). Thus, we estimate ${M}_{t^{*}}$ using ${\hat{M}}_{t^{*}}$ in the prediction model.

#### Definition 4

In the case of *irregular* (or intermittent) observations [13], ${\mathscr {D}}$ is processed to predict a soil moisture value ${\hat{M}}_{t^{*} + \tau }$ via a model *g*:

12 $$\begin{aligned} {\hat{M}}_{t^{*} + \tau }&= g({\hat{M}}_{t^{*}}, I_{t^{*}}, {\hat{I}}_{t^{*} + 1}, \ldots , {\hat{I}}_{t^{*} + \tau }), \quad \forall t^{*} > 0, \end{aligned}$$

where ${\hat{M}}_{t^{*}}$ has been compute recursively from previous data, previous estimates, and initial observation to ${M}_{0}$.

The irregular situation in (12) arises, for example, when measurements can only be performed during occasional field visits or by over-passing satellites or aircraft.

The regular and irregular approaches are illustrated in Fig. 6. With model inputs highlighted in circles (blue) and output in squares (green for regular and red for irregular), we show examples of forecasts given for regular and irregular measurements. In both cases, values at $t^{*} + \tau$, namely ${\hat{M}}_{t^{*} + \tau }$, are determined using one soil moisture input and $\tau$ rainfall observations or forecasts.

The key difference between the regular and irregular cases in Fig. 6 is the following. The true raw soil moisture observation, ${{\mathscr {D}}}_{t^{*}}$, is the input for the regular case, but the predicted value, ${\hat{M}}_{t^{*}}$, serves as the model input for the irregular case. The initial conditions, the same in both cases, are for simplicity not included in Fig. 6.

## Model analyses and forecast results

In our analysis, parameters of the mathematical models discussed in Sect. 4 were estimated using the process and data discussed in Sect. 3. Unless otherwise noted, our results reported in this section are for Canyon Fire field data.

Each model framework was initially applied to each moisture probe data at a given soil horizon without additional tailoring of the model parameters. That is, we sought models that could generally forecast soil moisture response irrespective of soil horizon. However, pilot experiments showed that a model, estimated for forecasting soil moisture at shallow depth, performs poorly in a deeper, more advanced soil horizon. Thus, model parameters were estimated for specific soil horizons, at different depths below the ground surface, in order to capture subtleties in timing and magnitude of soil moisture redistribution. Details about model analyses and forecasting results are found in the rest of this section.

### Metrics and measurements

To evaluate the quality of soil moisture forecasts, we measure three primary metrics in experiments: mean absolute percent error (MAPE), standard error, and maximum (absolute) error. MAPE reports the forecasting error in easy to understand percent scale:

13 $$\begin{aligned}&\text {MAPE} = \frac{100\%}{n} \sum _{t=1}^n \frac{| {\hat{M}}_t - M_t | }{ M_t}. \end{aligned}$$

Standard error provides us with a good measure of the goodness of fit by directly measuring the distance of data points from the regression line. Maximum error is particularly important in the context of soil moisture forecasting as the soil moisture peaks and valleys are critical in real-world applications (such as landslide warning system, or extreme dry soil indicator):

14 $$\begin{aligned}&\text {Max Error} = \max _{t=1, \cdots ,n} | {\hat{M}}_t - M_t |. \end{aligned}$$

Equations (13) and (14) define metrics over *n* observed and predicted soil moisture values. Methods with high errors near the peaks and valleys show high maximum error in absolute scale. These error metrics are often used in similar forecasting problems, such as in temperature profile prediction [56].

For all analyses and compute time measurements, except when noted otherwise, we used a 64 bit Linux computer with an Intel Xeon CPU 3.2 GHz processor.

### Optimization study

In general, there are no convexity guarantees in nonlinear regression. Hence, we use stochastic optimization algorithms to fit the models in (2), (4), and (6) to the training data. We experimentally study three general stochastic optimization algorithms: Real Coded Genetic Algorithm (RGA) [17], Simulated Annealing (SA) [7], and Differential Evolution (DE) [69]. We also experimentally study a statistical parameter tuning method IRACE [43]. We use R implementations of the algorithms discussed above, present in the R packages GA^9^ [67] (RGA), GenSA^10^ [76] (Generalized SA), DEOptim^11^ [48] (DE), and irace^12^ [43] (IRACE), respectively.

**Fig. 7:**
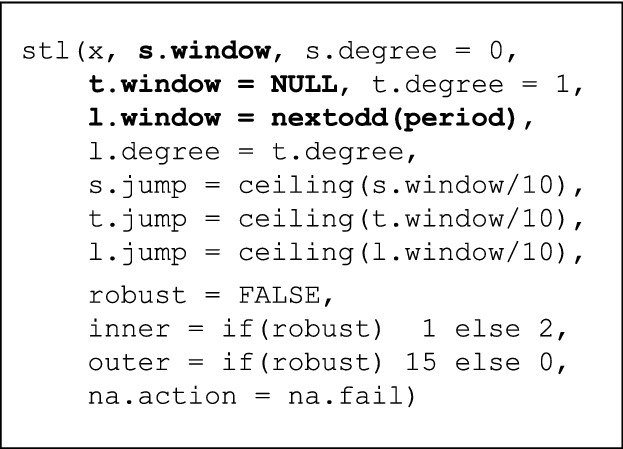
Input hyperparameters of STL’s R implementation as used in experiments, see Sect. 5.2

We use an R implementation of STL for preprocessing in our experiments [4].^13^ Figure 7 shows the R function definition of the STL implementation. Here, *x* is the input time series; values on the right-hand side of the hyperparameter arguments are default values. For our soil moisture datasets, we found that three free hyperparameters had major impact on the decomposition result. Highlighted in Fig. 7, these are seasonal window (s.window), trend cycle window (t.window), and the span of the loess window (l.window). To perform smoothing, we used HyperSTL^14^ to optimize STL hyperparameters by defining an objective (or fitness) function over the decomposed components [4].

With random initialization and parameters shown in Table 2, we ran each of the four optimization algorithms 30 times independently to find the AEAR and NAR parameters for the Canyon Fire 30 cm soil moisture dataset. The 30 cm soil moisture time-series poses significant challenges for forecasting and serves as a benchmark problem for our optimization study. The hyperparameter settings reported in Table 2 are a result of tuning experiments designed for efficient optimization. We set the prediction horizon to $\tau$ = 24 h for (3), (5), and (10). We recorded the prediction errors of the trained models on the test data along with the training time.

**Table 2:** Hyperparameter settings for stochastic optimization and statistical parameter tuning algorithms

Parameter	Algorithm			
	RGA	GenSA	DE	IRACE
Population size ($n_P$)	50	1	50	1
Num. generations ($n_G$)	100	5000	100	5000
Crossover prob. ($p_{CR}$)	0.8	N/A	0.5	N/A
Mutation prob. ($p_M$)	0.1	N/A	0.8	N/A

**Fig. 8:**
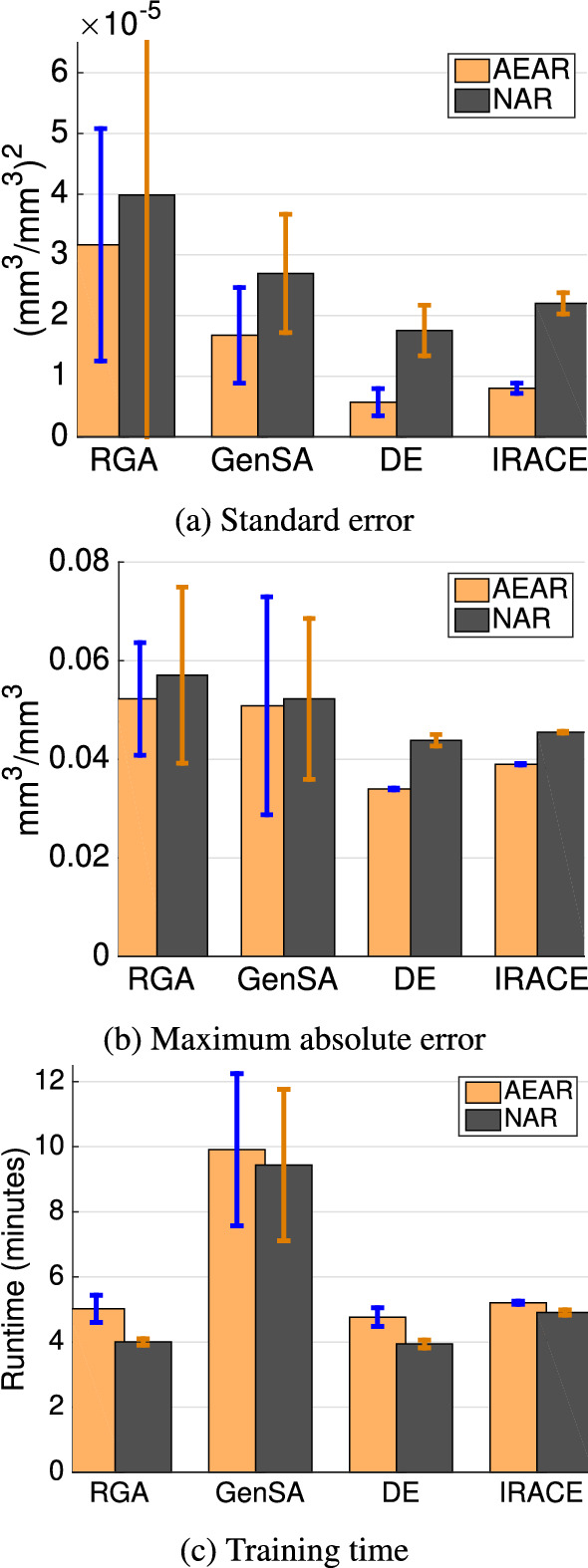
Results from 30 cm soil moisture data for AEAR and NAR models trained by four different optimization algorithms. Comparisons are of forecast error (**a**, **b**) and runtime of training (**c**). The mean and variance of all performance metrics are computed over 30 independent runs

Figure 8 shows comparative results, in the form of mean and standard deviation, for three performance metrics: standard error, maximum absolute error, and training time. Models trained by DE express minimum prediction errors. Also, the training time is consistently low for DE runs. Similar to previous results [4], we conclude that DE performs comparatively better than other optimization algorithms for this problem. Hence, we use DE to optimize the NAR and AEAR models in later experiments.

### Forecasting study

We compared the forecasting performance of the proposed models to several well-established and state-of-the-art baselines, including machine learning, time series, and soil moisture models. We provide a parallel comparison of the NAR and AEAR models to these baselines under the same evaluation metrics: Polynomial Regression (linear regression with polynomial features), Random Forest [8, 22], Support Vector Machine (SVM) [11], Auto-Regressive Moving Average Exogenous (ARMAX) [18], Long Short-Term Memory (LSTM) [23], and SEM [74]. We use the sklearn [57] implementation of Polynomial Regression, Random Forest, and SVM to forecast soil moisture with features being historical soil moisture and rainfall values similar to Eqs. 11 and 12. For ARMAX models, we use the *tsa* R package [12] and our LSTM models are implemented in PyTorch [55].

In this experiment, Canyon Fire soil moisture data at 30 cm depth are used for comparisons because it displays the most consistent, predictable behavior. Parameters *p* and *q*, in ARMAX(*p*, *q*), refer to the orders of autoregressive and moving average polynomials (see Sects. 5.5.1 and 6.2). In LSTM(*h*), the parameter *h* refers to the number of hidden units in the network. We use default parameters of the sklearn implementations of Polynomial Regression, Random Forest, and SVM.

Table 3 compares results for the baselines versus our NAR and AEAR models for two performance metrics: MAPE and maximum error. We report the mean and standard deviation of these two metrics over 30 independent runs. The trained LSTM(2), LSTM(4), NAR, and AEAR models have the smallest MAPE. The NAR and AEAR models are the only two models where MAPE is less than 10%. In addition, these models have an interpretability benefit, which we discuss in Sect. 5.8.

The Polynomial Regression, Random Forest, and SVM methods perform poorly in terms of both metrics and are not studied further. The remaining ARMAX, LSTM(8), LSTM(16), and SEM models do not appear as robust as NAR, AEAR, LSTM(2), and LSTM(4), but generally have lower errors than Polynomial Regression, Random Forest, and SVM. In terms of MAPE, LSTM(2) and LSTM(4) perform better than other LSTM models; LSTM(2) has the lowest standard deviation in Table 3 if we exclude Random Forest and SVM. However, the LSTM(2) model has a tendency for instability during steep rising and falling limbs of observed data. For example, Fig. 14 depicts pronounced mismatches between model forecasts and observed data during times of steep wetting and drying curves. Orland et al. [51] note similar problems during periods of heavy rainfall. As these are times of great importance for estimating landslide susceptibility, such model irregularities are problematic.

**Table 3:** Comparison of novel soil moisture models (NAR and AEAR) with classic machine learning models, state-of-the-art time series models, and soil moisture models with respect to Mean Absolute Percent Error (MAPE) and Maximum Error in forecasting soil moisture at 30 cm depth and with a 24 h time horizon

Forecasting model	MAPE	Maximum error ($\hbox {mm}^{3}\hbox {mm}^{-3}$)
Polynomial Regression	23.15% ($\pm \, 13.26\%$)	0.2341 ($\pm \, 0.1921$)
Random Forest	19.70% ($\pm \, 0.49\%$)	0.1901 ($\pm \, 0.0017$)
SVM	18.36% ($\pm \, 0.07\%$)	0.1660 ($\pm \, 0.0003$)
ARMAX(1,10)	17.83% ($\pm \, 9.53\%$)	0.0701 ($\pm \, 0.1706$)
ARMAX(5,10)	18.71% ($\pm \, 10.51\%$)	0.0782 ($\pm \, 0.1102$)
ARMAX(10,10)	18.13% ($\pm \, 10.79\%$)	0.0744 ($\pm \, 0.0984$)
LSTM(2)	9.8% ($\pm \, 4.95 \%$)	0.2046 ($\pm \, 0.1520$)
LSTM(4)	6.3% ($\pm \, 12.04 \%$)	0.1440 ($\pm \, 0.0210$)
LSTM(8)	13.95% ($\pm \, 7.38 \%$)	0.1511 ($\pm \, 0.1022$)
LSTM(16)	17.37% ($\pm \, 10.80 \%$)	0.1453 ($\pm \, 0.1030$)
SEM	19.25% ($\pm \, 14.16\%$)	0.1384 ($\pm \, 0.1720$)
NAR	7.83% ($\pm \, 11.73\%$)	0.0802 ($\pm \, 0.1494$)
AEAR	7.16% ($\pm \, 1.87\%$)	0.0597 ($\pm \, 0.0305$)

Mean and standard deviation of 30 independent runs are reported in each cell

The runtime comparison of training the above mentioned models reveals interesting properties for their practical use. These experiments were performed on a notebook connected to a CPU instance with an Intel Xeon Processor E5-2680 v4 and 50 GB RAM. Due to the high memory requirement of the Polynomial Regression method, we used for it a CPU instance with 200 GB RAM. Both Random Forest and SVM regression methods take 8–15 s for each training run. ARMAX models take 1–5 min. LSTM models take 3–17 min, the SEM model takes 20–60 s, and our NAR and AEAR models take 4–6 min. The training time of LSTM models increases with the number of hidden units. On the other hand, our NAR and AEAR models’ runtime is primarily determined by the optimization algorithm. Separate from all, the Polynomial Regression takes over 30 min and a large amount of memory. Polynomial Regression is prohibitively slow to train and is therefore of less practical value as compared to other methods. While Random Forest, SVM, and the SEM models take little training time, the errors in predicted soil moisture values are higher than for other models. The LSTM, NAR, and AEAR models take moderate training times and produce accurate forecasts.

As a result of the comparative study above, we focus our analysis in the rest of this section on improving the understanding of the performance of ARMAX, LSTM, and SEM versus our AEAR and NAR models.

### Irregular or intermittent measurements: NAR and AEAR

The NAR model was fit separately to the 5 and 15 cm data. Although NAR fits the shallow moisture data (5 cm) quite well, it generates substantial forecast misfits for the deeper levels of 15 and 30 cm (not shown as figure). Apparently, soil moisture variations at deeper levels do not resemble a simple exponential curve. As such, the approach is inconsistent with soil moisture response at deeper levels, which likely reflects additional water transported through the soil column as rapid macro-pore flow, topographically focused water from the above contributing drainage area, or possibly as return flow from underlying bedrock.

**Fig. 9:**
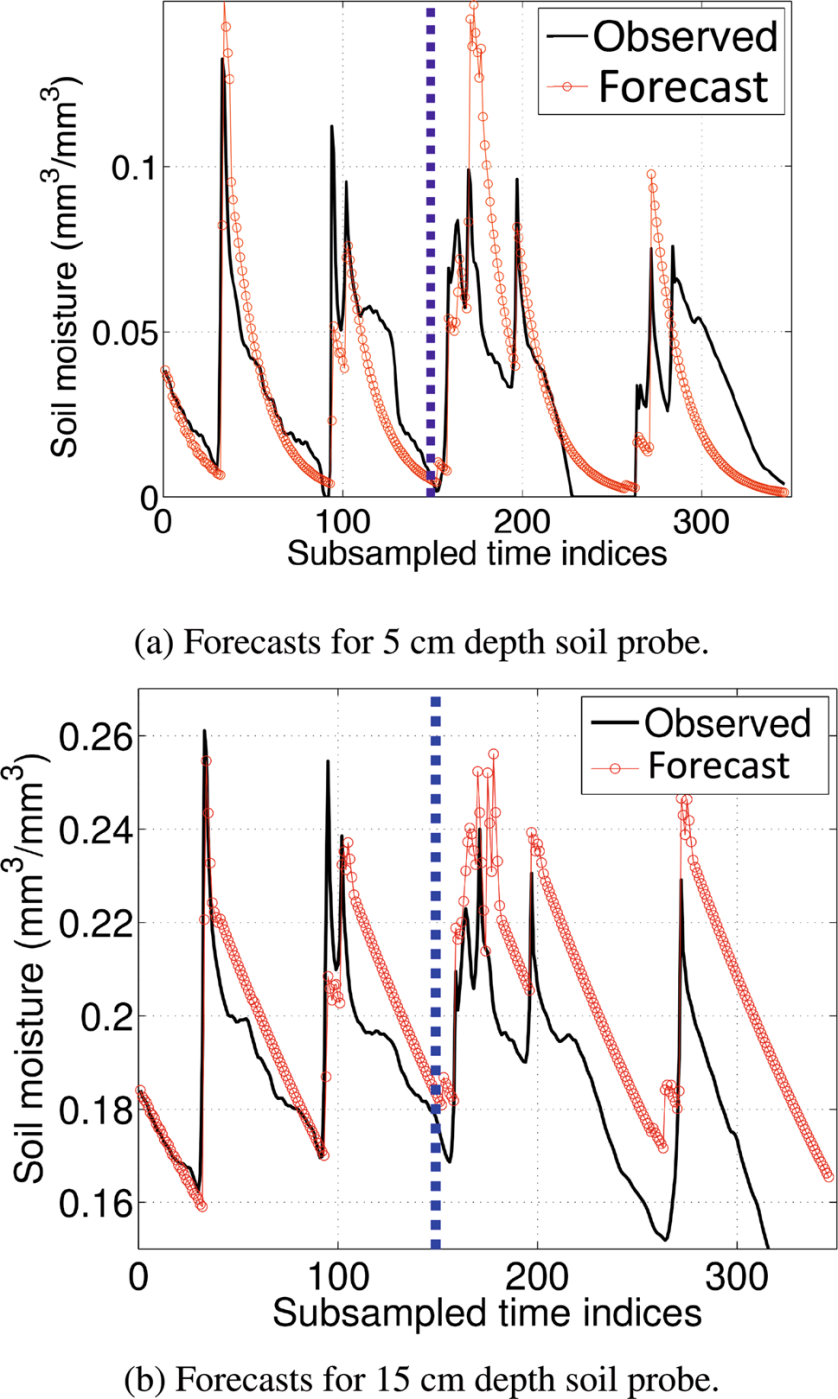
Soil moisture forecasts for 5 and 15 cm depths using the AEAR model, based on a one-point measurement of soil moisture at $t=0$. The points to the left of each of the blue dashed vertical lines are in the training set $\mathscr {M}_{\mathscr {T}}$, and the points to the right are in the test set $\mathscr {M}_{\mathscr {P}}$

**Fig. 10:**
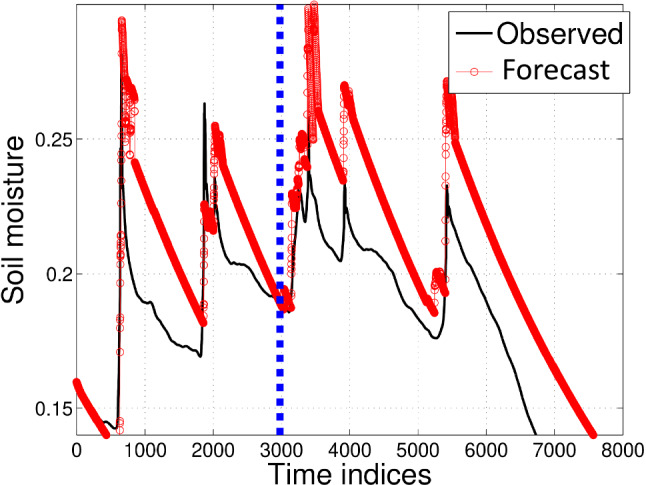
Moisture forecasts for 30 cm depth using AEAR model. Points to left of blue dashed vertical line are in the training set $\mathscr {M}_{\mathscr {T}}$ and on the right in forecasting test set $\mathscr {M}_{\mathscr {P}}$

When estimating the AEAR parameters from (10), the constraint $k_s > k_g$ was implemented by introducing a ratio $k_g/k_s$ and setting $k_g/k_s < 1, k_s > 0$ during optimization. Predicted values for the 5 and 15 cm levels using trained AEAR models are shown in Fig. 9. The AEAR model fits the 5 cm data equally well as the NAR model, but gives much better forecasts for the 15 cm case. The NAR model tends to underpredict soil moisture at deeper depths. Hence, the AEAR model appears to perform better in soils prone to fast water transmission arising from preferential flow paths. The AEAR forecast for the deepest level, 30 cm, is presented in Fig. 10.

### Regular and irregular measurements: forecast results

We compare the SEM, NAR, and AEAR models for both regular (11) and irregular (12) measurements, as well as with classical time series forecasting methods. We use an autoregressive model as a baseline time series forecasting method [2]. In particular, we use an autoregressive moving average model with exogenous inputs (ARMAX), accounting for precipitation as exogenous input terms. We study three ARMAX models of increasing complexity.

**Fig. 11:**
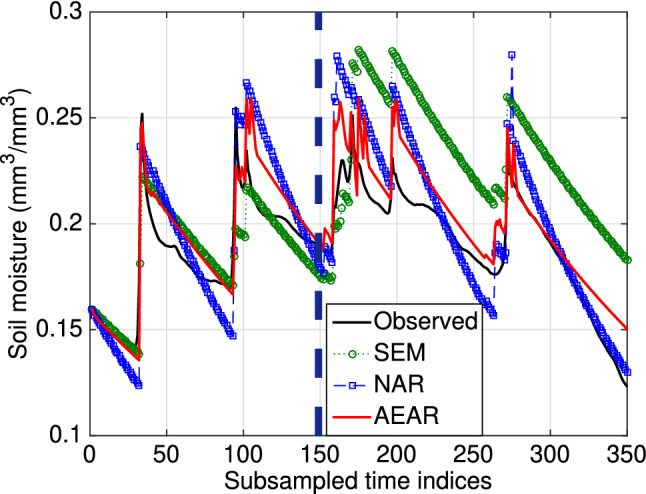
Comparison of different model forecasts (SEM, NAR, AEAR) at 30 cm depth. The standard error in these forecasts is $0.037, 0.022, \text { and } 0.020$, respectively. The vertical blue dashed line separates the training set $\mathscr {M}_{\mathscr {T}}$ (to the left) and forecasting set $\mathscr {M}_{\mathscr {P}}$ (to the right)

**Fig. 12:**
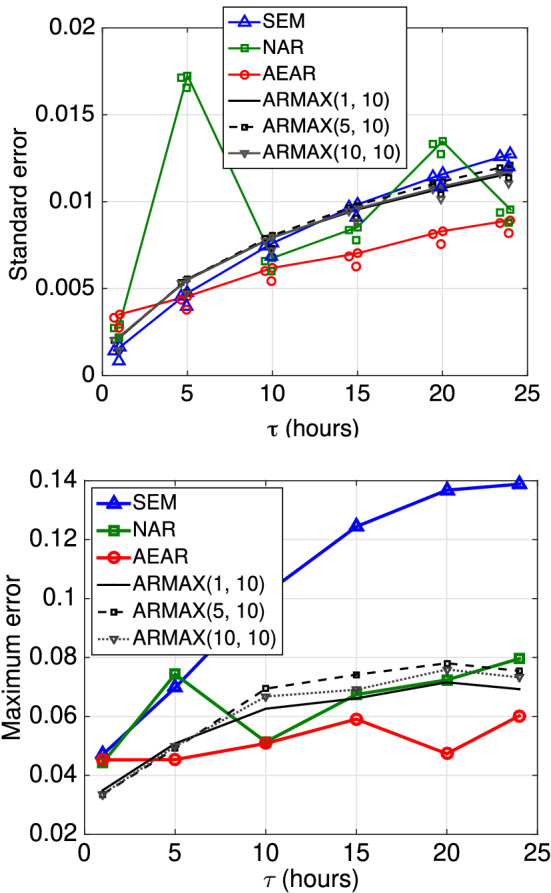
Forecast errors as a function of prediction horizon ($\tau$), varying on the *x*-axis, for regular measurements. Top: Standard error is on the *y*-axis. Bottom: Maximum error is on the *y*-axis

#### Regular measurements: various models

The time horizon $\tau$ is a critical parameter in time-series forecasts with regular measurements. We trained SEM, NAR, and AEAR models and three ARMAX models with increasing time horizons $\tau =$ 1, 5, 10, 15, 20, and 24 h.

Assuming equal availability of historic soil moisture and rainfall data, we keep the orders of the autoregressive and exogenous input polynomials (16) the same, i.e., $p = b$. We suppress the third parameter of ARMAX(*p*, *q*, *p*) model and denote it as ARMAX(*p*, *q*). With the R implementation of ARMAX methods within *tsa* package, we use standard system identification methods [42] to learn the parameters *p* and *q* from our training data.

We present the error on the test data of trained models for different forecast horizons ($\tau$) in Fig. 12, which illustrates a key message of this paper. Clearly, the standard error of existing SEM model is smallest for $\tau = 1$; however, for the short time horizon, all the models perform quite well. The more complicated task is forecasting for longer time horizons $\tau$, and SEM’s standard error increases with $\tau$ at a much higher rate than AEAR’s error. The ARMAX models show similar error-growth as the SEM model. For greater time horizons, $\tau \ge 10$, our AEAR model shows minimum standard error among all models.

A clear divergence between the maximum errors (with increasing $\tau$) of the existing SEM model and the accumulative rainfall models (NAR and AEAR) is observed to the right in Fig. 12. The maximum error of the SEM model exceeds 0.12 at $\tau = 15$. As the variations in soil moisture in our dataset lies within 0.3 $\hbox {mm}^{3}\hbox {mm}^{-3}$, more than 0.1 $\hbox {mm}^{3}\hbox {mm}^{-3}$ off forecasts imply higher than 33% error. Moreover, a 0.3 $\hbox {mm}^{3}\hbox {mm}^{-3}$ difference in soil moisture suggests very different soil conditions [15]. The AEAR maximum error curve, in contrast, is much flatter and it remains within 0.06 mm$^3$ mm$^{-3}$ while forecasting soil moisture values a day ahead ($\tau = 24$). This indicates that the AEAR model is capable of forecasting soil moisture to time horizons of $\tau = 5$ to $\tau = 24$ h, another major modeling improvement.

Although the ARMAX models perform better than the SEM and NAR models in terms of maximum absolute error, the error growth rates are higher than for AEAR. Hence, the AEAR model, with parameters directly related to hydrological processes, shows superiority over more complicated ARMAX models with a higher number of parameters.

#### Irregular measurements: various models

A comparison of trained SEM, NAR, and AEAR models with 30 cm soil moisture data is shown in Fig. 11. The irregular setting is not appropriate for ARMAX models, due to high computational complexity of model fitting. Therefore, we do not compare with ARMAX here, unlike what was done for the regular measurements.

Although the existing SEM model fits the training data $\mathscr {M}_{\mathscr {T}}$ fairly well, it does not adequately represent the moisture variation in the test data $\mathscr {M}_{\mathscr {P}}$. The NAR model attempts to find an average exponential model around the observed variations. In contrast, the AEAR model neither overfits the training set, nor deviates from the observed moisture substantially in the test data. The forecast error of the AEAR model at the moisture peaks is bounded by 0.04 $\hbox {mm}^{3}\hbox {mm}^{-3}$, which falls within the range of typical accuracy of various volumetric water content sensors using a factory calibration [28, 35]. The AEAR forecast closely follows the observed soil moisture values. Other models, namely NAR and SEM, produce greater mismatch for both the peaks and valleys. Since peak soil moisture values have important practical applications, *e.g.,* predicting landslides, the AEAR model is best suited to scenarios where errors in peak soil moisture should be minimized.

### Regular measurements: AEAR and LSTM

Due to the recent focus on neural network research, many complex architectures have emerged for time series forecasting. In this paper, we consider a typical LSTM sequence network architecture as a benchmark model [23, 37, 51]. Our goal is to experimentally study the merits and limitations of a sequence model with many more parameters as compared to the AEAR or NAR models.

We pass a rainfall sequence (of length $\tau + 1$) and historic soil moisture value as inputs to LSTM units. The LSTM output is connected to a hidden layer with Rectified Linear Unit (ReLU) activation. The number of hidden units is varied to find the most suitable architecture for soil moisture forecasting.

The LSTM architecture is implemented using PyTorch [55]. The network is comprised of input LSTM units to encode the sequence of soil moisture and rainfall values, and fully connected dense layers to produce the final continuous output. We feed the concatenated array of soil moisture and rainfall values to the LSTM layer with *h* hidden units. The output of LSTM is passed through a fully connected hidden layer with 128 neurons. This layer maps the *h*-dimensional LSTM output to 128 dimensions. Finally, we pass the output of the hidden layer through a fully connected output layer to produce single continuous output. The output of LSTM and hidden layers have ReLU activation. To study the effect of model complexity in soil moisture forecasting, we vary *h* and fit the network parameters using the *Adam* optimizer [34].

**Fig. 13:**
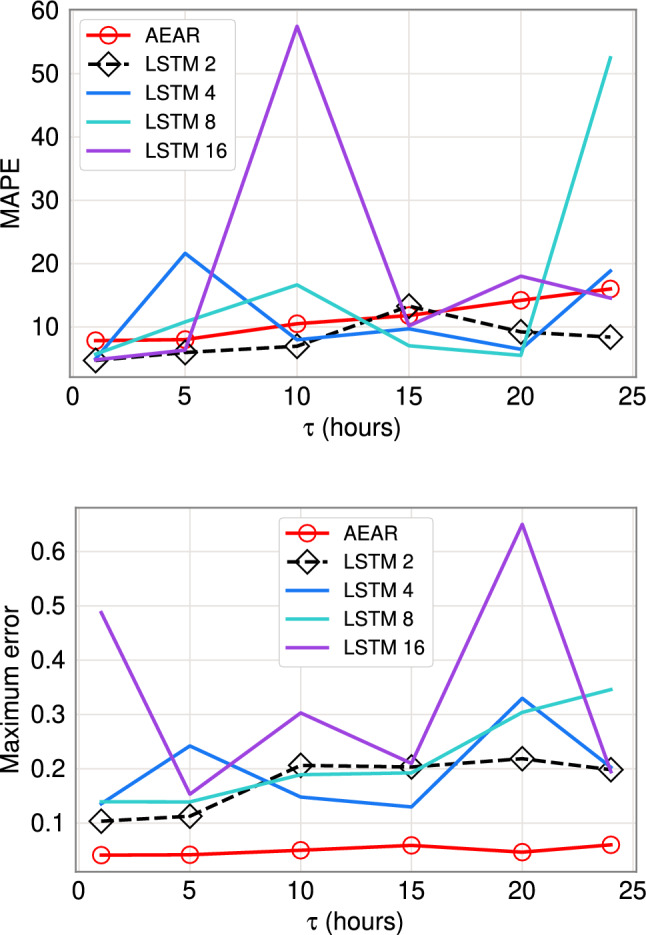
Forecast error for a 30 cm probe as function of prediction horizon ($\tau$), varying on the *x*-axis, for regular measurements. Top: MAPE on the *y*-axis. Bottom: Maximum error on the *y*-axis

**Fig. 14:**
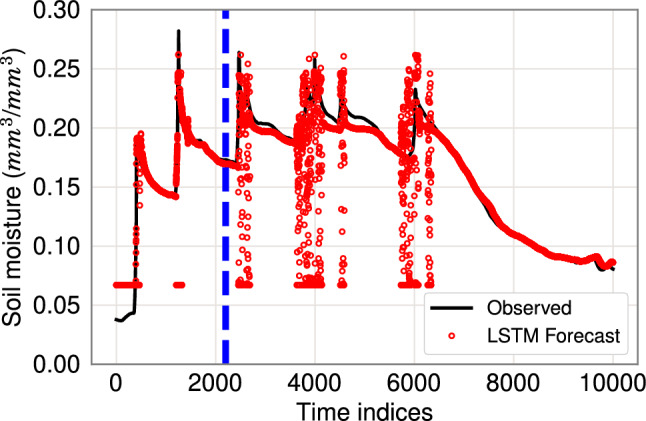
Soil moisture forecasts at 30 cm depth using an LSTM(2) model in the regular setting. Points on the left of blue dashed vertical line are in the training set $\mathscr {M}_{\mathscr {T}}$ and on the right in the forecasting test set $\mathscr {M}_{\mathscr {P}}$

We use LSTM(*h*) models in the regular setting according to Eq. 11. We keep the same train-test partition as in the case of evaluating NAR or AEAR models. To find the best architecture, we vary *h* and train different models for 30 cm soil moisture forecasting. Figure 13 depicts the variation in standard error (or mean squared error), maximum error, and mean absolute percent error (MAPE) with increasing prediction horizon $\tau$. The same figure also shows the comparison with AEAR model. We see that LSTM(2) and LSTM(4) perform better than the other LSTM models. Therefore, we show the soil moisture forecast of one top candidate, LSTM(2), in Fig. 14, where the vertical blue line separates train and test data. LSTM(4) produces very similar forecast.

Figure 14 shows the LSTM(2) model forecasting the drying regions accurately, leading to low standard error and MAPE, which reflects total forecast error. However, near high rainfall storminess, there are forecast spikes, leading to high maximum error. While the AEAR model yields accurate forecasts for both wetting and drying responses of soil moisture, the LSTM model performs particularly well for the drying response. This is consistent with the LSTM(2) and AEAR results in Table 3. Similarly, Orland et al. [51] note how LSTM poorly forecasts hydrologic response during continuously wet intervals. Therefore, a possible refinement is to combine AEAR and LSTM to achieve more robust forecasts.

### Controlled experiment bucket data: forecast results

We now consider data resulting from the controlled experiment and compare models using regular measurements. Among the ARMAX models, we choose ARMAX(10, 10), which performed best for the Canyon Fire dataset.

**Fig. 15:**
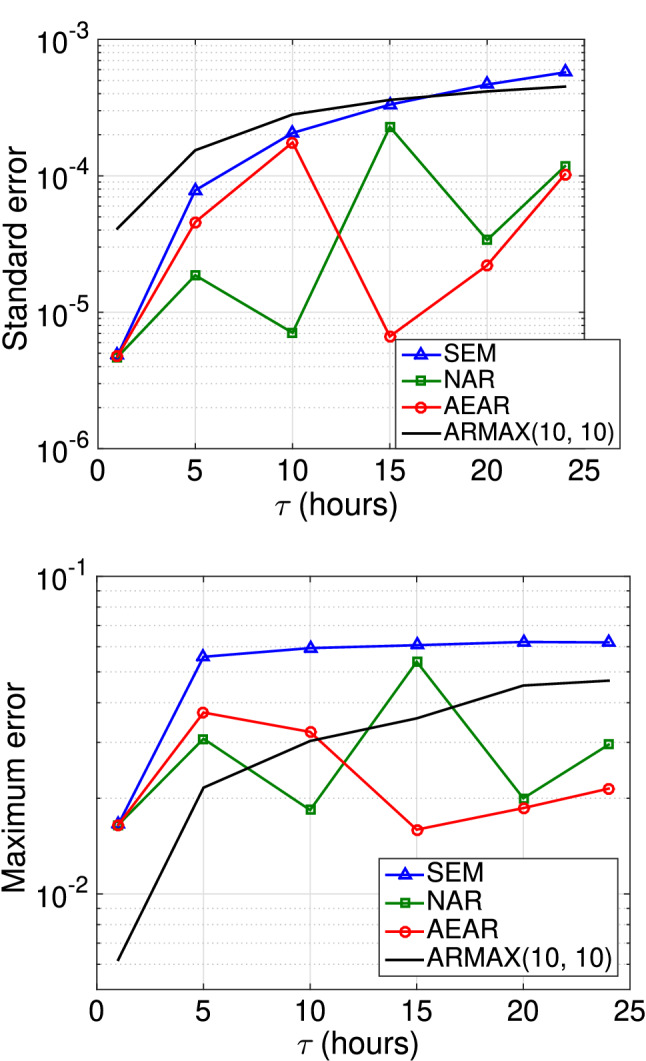
Results for controlled experimental bucket data. Forecast errors for different models are on the *y*-axis, as a function of varying $\tau$ (in regular measurements) on the *x*-axis. Top: Standard error on the *y*-axis. Bottom: Maximum error on the *y*-axis

Similar to the previous section with regular measurements (Sect. 5.5.1), we use the trained SEM, NAR, AEAR, and ARMAX models to forecast the soil moisture at 15 cm depth. Figure 15 shows the standard and maximum absolute errors of all four models for the test dataset. The NAR and AEAR models express standard errors lower than those for the SEM and ARMAX models for the $\tau$-values of greatest interest. Although ARMAX shows low maximum error for $\tau = 1 \text { and } \tau = 5$ h, for longer forecast horizons $\tau \ge 10$ h either one or both of the NAR and AEAR models achieve lower error. For shorter time horizons, 1–10 h, the NAR model produces more accurate overall forecasts relative to other methods in terms of standard error. The AEAR forecasts are most accurate for $\tau \ge 15$ hours, for both standard and maximum errors.

The SEM model forecasts have a smaller increase in maximum error for longer forecast horizons ($\tau = 24$ h), as compared to Fig. 12. This reaffirms how field-based soil moisture includes three-dimensional moisture flow that is more complicated than in controlled experimental settings.

### Validation of model interpretability by earth scientists

Both the AEAR and NAR models are intended to be interpretable by earth scientists, to represent hydrologic processes in soil and fit the soil moisture data well (see interpretability requirement in Sect. 2.2). The experimental results earlier in Sect. 5 suggest that AEAR better models the data across multiple soil layers and also gives better forecasts compared to NAR. Consequently, we recommend the AEAR model and now explore estimated AEAR parameter variability with season and soil depth for Canyon Fire data. Parameter disparities may reflect the spatial variability of soil wetting and drying cycles, hysteresis of additive cycles, and changing ground surface conditions from erosion and redeposition of mobile sediment following storms.

Orland et al. [51] present a forecasting approach for landslide prone hillslopes. They use soil moisture, rainfall, and pore-water pressure to train an LSTM model that forecasts suction (pore-water pressure) over 36 hr time intervals. Their approach is similar to ours, but with a few crucial differences: (i) although they use soil moisture in model training their published forecasts are for suction only, not soil moisture; (ii) their LSTM approach does not result in physically based parameters necessary to understand the hydrologic process; (iii) their LSTM model has poor robustness, with occasional high LSTM errors that appear to be due to forecasts spanning a wide range relative to the measurements around major rainfall events (see the “vertical stripes” of red dots in Fig. 14 in our results, starting right after the blue dashed line around time index 2000); and (iv) the Oregon landscape they use data from is quite different from our Canyon Fire setting. Below we provide further details regarding Point (i) and Point (ii), emphasizing how our results provide a deeper insight into the soil moisture wetting and drying process.

#### Variation in soil properties over time

**Fig. 16:**
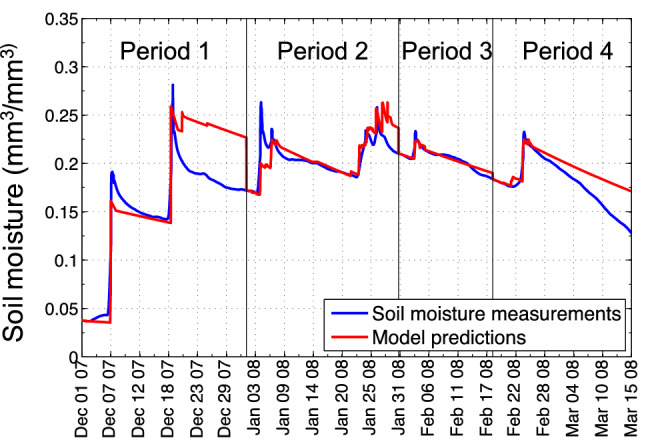
Partition of Canyon Fire soil moisture data from 30 cm deep probe measurements and forecasts of trained AEAR models for each period. To compare the model parameters for different periods, we partitioned the almost four months of the winter 2007–2008 season (December–March) into Period 1 (“December”), Period 2 (“January”), Period 3 (“February”), and Period 4 (“March”)

Immediately following the Canyon Fire, the soil was extremely dry from the passage of fire. After two rain events in December 2007, the soil properties of initial moisture content prior to rainfall and infiltration rate changed significantly. Thus, the moisture response for the first two rainfall events, which eroded the surface and transported ash and char downslope, is hypothesized to be too different to be captured by one average model. Although the storm of 18–19 December 2007 triggered multiple small but long-traveled debris flows (Fig. 2), none of the following storms of similar rain intensity and duration relations, as depicted in Fig. 3, triggered any observable debris flows. There was, however, notable ground surface change resulting from surface erosion and sediment transport in response to each successive rain storm. To isolate the non-stationary behavior of ground surface conditions over time [13], we partitioned the data into four time periods, see Fig. 16, bracketing rainfall from storms and related increases and subsequent decreases in soil moisture. Each period covers about one month. However, Period 3 (“February”) ends early, in order not to interfere with a storm event near February 23.

**Fig. 17:**
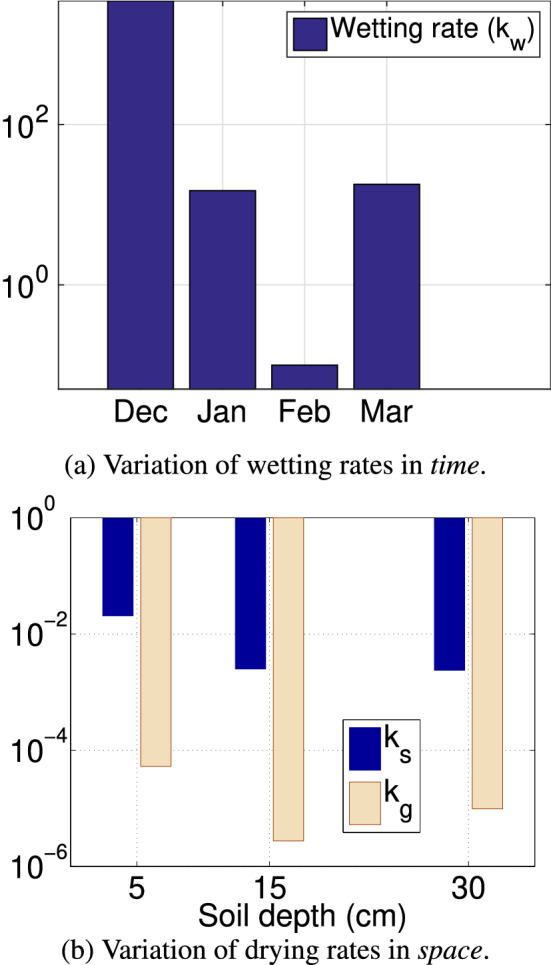
Variation in wetting and drying properties of soil in AEAR model parameters. We depict the variations over a winter season’s data, shown in Fig. 16, in both *time*—different wetting rates over “months,” as shown in (**a**) and *space*—drying rates at three different depths, as shown in (**b**). To observe the spatial variation, the model parameters for various soil depths below ground surface are shown in (**b**)

To study the variation in model parameters over time, we trained separate AEAR models for each time period, see Figs. 16 and 17. A large variation in the model’s wetting rate $k_w$ is observed in the four periods following the wildfire (Fig. 17a). At the beginning of Period 1, extremely dry soil absorbs rain almost instantaneously, leading to a very high wetting rate $k_w > 100$. As soil pores fill with water and illuviated fine-grained sediment, the wetting rate $k_w$ decreases and eventually goes to $k_w < 1$ in Period 3. Later in the season, $k_w$ increases in Period 4 (late-February to mid-March) as the soil starts drying, atmospheric temperatures rose, and post-fire vegetation regrowth decreased soil moisture by increased evapotranspiration.

#### Soil moisture variation with soil depth

The timing and magnitude of soil drying at different depths vary due to environmental factors such as solar radiation penetration, air temperature, wind speed, and water absorption by vegetation. The trained AEAR parameters $k_s$ and $k_g$ for models from three different soil depths can potentially capture these effects. Figure 17b shows the empirical variation in $k_s$ and $k_g$ with soil depth. Here we trained three different models for the three soil depths using training data from December 2007 to March 2008. We observe the drying rates of the shallowest soil horizon (5 cm) are much higher compared to the deeper, more pedogenically mature soil horizons. Younger, coarser textured soils near the ground surface are directly exposed to higher solar radiation, and atmospheric effects, such as wind, increasing relative evaporation. Moreover, $k_s$ and $k_g$ differ by at least two orders of magnitude consistently over all soil depths. This suggests that our introduction of distinct drying terms in the AEAR model (Eq. 6) is well-justified.

## Related work

We now examine models that seek to meet our goals and requirements discussed in Sect. 2. Here we consider methods from different disciplines–machine learning, earth science, and statistical modeling.

### Simple exponential model (SEM)

The Antecedent Water Index (AWI) presented in equation (2) is considered to be proportional to soil moisture and is widely used to model soil moisture response [16, 74]. The AWI model is inspired by a water balance equation (15):

15 $$\begin{aligned} \Delta M^S_t = I_t - E_t - R_t - G_{t}, \end{aligned}$$

where $\Delta M^S_t$ is the change in soil water content at time *t*; $I_t$ is the mean precipitation; $E_t$ is the mean evapotranspiration; $R_t$ is the net streamflow divergence; and $G_t$ is the net groundwater loss.

Essentially, (15) balances precipitation, evapotranspiration, and water loss to the rate of change of soil moisture. It has been shown that an approximation of (15) leads to an exponential decay model (under no precipitation) [25], which is very similar to the AWI model (2).

NOAA forecasts of soil moisture use surface hydrology as defined by (15) [73]. Hence, the AWI model is closely related to a state-of-the-art hydrology-inspired soil moisture model. The AWI model was also used to forecast shallow landslides from rainfall in Seattle, Washington [16].

### Statistical models for time series forecasts

We now consider traditional time series models from statistics, namely ARIMA, ARMA, and ARMAX. Aljoumani et al. [2] investigate the impact of irrigation on soil water content in a silty loam soil using an autoregressive integrated moving average (ARIMA) model. Under the normality assumption, the ARIMA model could not properly explain the effect of variable interval irrigation. To remedy this problem with the ARIMA model, outlier detection and intervention analysis were used. Unfortunately, their model is hard to interpret from the perspective of physical soil processes.

Khaertidova and Longobardi [33] analyze soil moisture dynamics in inter-storm periods to deduce that moisture reduction magnitudes are seasonally dependent with highest rates corresponding with plant water uptake. Given their focus on inter-storm periods rather than seasons or years, there is no analysis of soil moisture increases and a simple exponential decay model is sufficient.

In hydrologic literature, various autoregressive models exist for data-driven rainfall–runoff modeling [58]. When soil properties and water transport do not behave as random functions, but rather as structured processes, ARMA (autoregressive moving average) and ARIMA models can be used to model soil moisture dynamics [2, 10].

An ARMAX(*p*,*q*,*b*) model expresses the dependence of past soil moisture values $\{ M_{t-i} \}$ and rainfall $\{ I_{t-l} \}$ on present soil moisture $M_t$ according to the following equation:

16 $$\begin{aligned} M_t = \sum _{i=1}^p \phi _i M_{t-i} + \sum _{j=1}^q \theta _j \epsilon _{t-j} + \sum _{l=1}^b \eta _l I_{t-l}, \end{aligned}$$

where *p*, *q*, and *b* represent the time-delay in autoregressive, moving average, and exogenous input terms, respectively. And $\phi _i,$ $\theta _j, \text { and } \eta _l$ are their corresponding weights. Here $\epsilon _{t-j}$ represents white noise and is a moving average model of order *q*.

In our experiments in Sect. 5, we typically keep the orders of the autoregressive and exogenous input polynomials (16) the same, i.e., $p = b$. We therefore can abbreviate ARMAX(*p*,*q*,*b*) as ARMAX(*p*,*q*).

### Decomposition methods for time series

Time series decomposition methods perform analyses by splitting up (or decomposing) the signal. One such method is known as seasonal decomposition of time series by Loess (STL). STL is a seasonal trend decomposition method that uses local regression [9]. STL decomposes a time series into trend, seasonal, and remainder components. STL has been used in several areas of science and engineering, for example, to analyze seasonal patterns in suicides [54] as well as for earth science time series data analysis [4].

STL and other existing methods, such as moving averages, may fail to smooth time series data while preserving peaks and valleys [4]. Therefore, we developed a novel method, HyperSTL, for extrema-preserving smoothing by optimizing the parameters of STL [4]. HyperSTL successfully reduces noise, including instrumental variations, while preserving extrema and signal detail. We demonstrated our method on post-fire soil moisture time-series data [4] and also apply the method in Sect. 3.2.

### Regional soil moisture models

The 3-layer Variable Infiltration Capacity model (VIC-3L) is widely used to simulate global soil moisture [71]. The VIC-3L model was developed as a generalization of the single layer VIC hydrological model [40]. It incorporates many parameters accounting for sub-grid variability in soil moisture, land surface vegetation, precipitation, and topography. The Palmer Drought Severity Index (PDSI) and Standardized Precipitation Index (SPI) are also used to estimate soil moisture over large spatial and long temporal scales [68].

VIC-3L, PDSI, and SPI and similar regional approaches are applicable over broad areas, but not appropriate given our requirements. These indicators are not appropriate for either the spatial or temporal scales of local erosion and shallow landslide processes. A primary limitation of the PDSI is that it cannot be correlated with site-specific water resources such as soil moisture or runoff. Rather, it is a unitless generalized index applicable at the spatial scales of states or counties. Both the PDSI and the SPI rely on data at monthly intervals for long-term (monthly to yearly) assessments of available moisture, whereas we are interested in timescales of hours to evaluate local runoff and heightened soil moisture and positive pore-water pressure generation in the context of shallow landslide susceptibility.

### Artificial intelligence and machine learning methods and models

Several studies review, benchmark, or compare statistical and machine learning methods, sometimes leading to controversial or surprising conclusions [20, 21, 37, 45, 53]. For example, Makridakis et al. experimentally compare statistical and machine learning forecasting methods to conclude that on the time series they experiment with, the statistical methods have higher accuracy than the machine learning methods [45]. Later, Papacharalampous et al. compare different methods to forecast hydrological processes. In contrast with Makridakis et al., they conclude that stochastic methods, including several statistical methods, and machine learning methods produce equally useful forecasts [53]. Hewamalage et al. reach similar conclusions [21]. These results have led to continued focus on both statistical and machine learning methods for time series forecasting. Such time series forecasting research includes, in the case of machine learning, investigations of neural networks including deep learning for time series forecasting [37]. Deep learning architecture experiments with times series data indicate that Long Short-Term Memory (LSTM) models can achieve the most accurate forecasts [37, 51].

In many cases, time series data contain contextual information that is long-range. Such long-range contextual information may be very important when mapping between input and output sequences. For many recurrent neural network (RNN) architectures, the range of the context that can be handled is very limited. Research on this problem has been going on for decades; however, a gradient-based RNN approach called Long Short-Term Memory (LSTM) developed by Hochreiter and Schmidhuber [23] makes substantial progress on solving the long-range contextual information problem. The LSTM architecture efficiently models long-term dependencies of time-series and does not suffer from the vanishing gradient problem [63]. These properties of LSTMs make them useful for time series forecasting in earth science, for example, for sea surface temperature forecasting [41] and forecasting of pore-water pressure in landslide-prone hillslopes [51]. Employing LSTMs, Orland et al. [51] forecast the timing and magnitude of pore-water pressure, as opposed to soil moisture, at 36 hr intervals. Their LSTM approach alone, though, does not result in a deeper comprehension of the physical processes driving hydrologic response. Here we apply LSTM as a benchmark time-series forecasting algorithm and present comparisons with our proposed methods in Sect. 5.6.

There is currently broad discussion and interest around trustworthy, explainable, interpretable, comprehensible, fair, transparent, and understandable models and algorithms in artificial intelligence and machine learning [39, 47, 49, 62]. Whereas there is general agreement on concept importance, inconsistency and confusion remain in the precise meaning of the terminology. In particular, the terms “explainable” and “understandable” can be applied to models developed here. “Explainable machine learning” has gradually become most associated with explaining deep neural networks and explaining similar black box models with millions or billions of parameters. Although important and interesting, the topic of explaining black box models is not the focus here. Consequently, we prefer the term “interpretable ML” or “interpretable model,” consistent with Rudin’s usage in the context of models aiding high-stakes decision making [62]. Similarly, Murdoch et al. highlight “model-based interpretability” in their review of definitions, methods, and applications in interpretable machine learning [49] related to human health and safety [39, 62].

Related to the focus on interpretable models is the combination of physics-based models with complex data driven approaches including neural networks. A physics-based neural network, for example, was used to model lake temperature evolution [29].

Evolutionary algorithms (EAs) and stochastic optimization algorithms have been successfully employed in many hard optimization problems, including machine learning problems, without introducing strong assumptions such as convexity. Examples of such EAs include Real Coded Genetic Algorithm (RGA) [17], Simulated Annealing (SA) [7], and Differential Evolution (DE) [69]. In DE, the difference vector-based mutation and the uniform crossover schemes are favorable for exploring patterns in the search space. DE is, for example, used for parameter estimation of non-linear models [75]. SA is a probabilistic search technique, and in contrast with DE and RGA it is not population-based. To prevent getting stuck at local optima, SA accepts random neighbors with a small probability. SA-based optimization methods were used for several time series forecasting problems including electricity load forecasting and traffic flow estimation [24, 38, 52]. SA has also been successfully used to solve many difficult data mining problems, such as influence maximization in social networks [26].

In artificial intelligence, including machine learning, a need often exists to optimize hyperparameters of complex algorithms. IRACE performs iterated racing to automatically tune parameter configurations of an algorithm. IRACE has been successfully used for time-series analysis [1] and feature selection [78]. HyperSTL augments STL [9] with a stochastic optimization algorithm, a genetic algorithm, to tune the hyperparameters of STL (see Sect. 6.3 and Fig. 7). HyperSTL is controlled via an objective function with three terms that formalize extrema-preservation: RMSE with a straight line to smooth the trend line *T*; range of remainder *R* from STL to minimize high and low peaks; and variance of remainder *R* to reduce seasonality detection. These three components were combined into a weighted sum, which was then minimized by HyperSTL. The HyperSTL technique enables the identification of both short- and long-term seasonality in soil moisture datasets while retaining peaks and valleys [4].

### Evaluation of requirements

We now evaluate the methods and models discussed above in light of the problem of soil moisture forecasting and the requirements set forth in Sect. 2.

Although soil moisture is of critical importance to many disciplines, widespread in situ soil moisture monitoring at high-frequency (period of minutes) has only recently become commonplace. Further, Kean et al. discuss the short timeframe of responses for post-fire sediment-laden floods and debris flows, necessitating the collection of relatively high frequency data [31]. The best correlation between flood peaks and rainfall in Southern California was determined to be $<30$ min, with the 5-min rainfall intensity being the most accurate metric [31]. Consequently, the challenges of analyzing and forecasting soil moisture present an ideal opportunity to take advantage of recent advances in data science, machine learning, and statistical time series analysis.

While doing so, it is important to keep in mind the three requirements outlined in Sect. 2.2. The SEM discussed in Sect. 6.1 has the potential to meet all these requirements. It meets two of the requirements very well (being interpretable and data-driven) but is not as accurate for medium-term forecasting (the third requirement) of importance to public safety and agricultural needs. The other methods discussed above do not appear as promising. Therefore, the decision was made to take the SEM as a starting point and develop it further as discussed in Sects. 3 and 4 in order to improve medium-term forecasting accuracy while not compromising significantly along the two other dimensions of being data driven and interpretable.

## Conclusion and future work

### Conclusion

Time-series data represent some of the most important forms of information chronicling the health and trajectory of Earth and her biological systems. In recognition of the importance of monitoring environmental data in parallel with technology advances for doing such monitoring, the quantity and variety of time-series data are rapidly expanding. These datasets represent critical information for work in earth science. As a major provider of widely ranging earth science time-series data to the public, the USGS plays a critical role in not only collecting such data but in providing cutting edge tools for interpreting these complex data.

In order to better analyze often diverse and complex data, the cross-disciplinary project reported here has introduced machine learning approaches to existing earth science data and analysis capabilities. The focus has been on forecasting soil moisture with greater accuracy compared to existing methods.

We modeled soil moisture response with respect to rainfall from natural storms and determined that the existing SEM (AWI) approach has several strengths but does not adequately forecast moisture response for time periods greater than 5–10 h, depending on the dataset. Moreover, it does not perform well for deeper soil horizons in a post-fire setting, because soil moisture variations in the deeper horizons do not follow a simple exponential curve.

Building on the SEM framework, we developed two novel soil moisture models, NAR and AEAR, that are rooted in deterministic, physically based hydrology. The simpler NAR model often generalizes well for shallow soil depths and shorter forecast horizons ($1 \text { hour } \le \tau \le 10$ h). Alternatively, the AEAR model works well for moisture data from three different soil layers with distinct soil textures and over longer time horizons. Our AEAR model can be trained with both regular and irregular time series measurements of soil moisture and provides good forecasts in both cases. Moreover, AEAR inherently represents hydrological soil processes, as validated by earth scientists, providing a means to estimate rates of soil wetting during rain storms and drying in-between storms for varying soil depths with distinct pedogenic horizons. In the context of forecasting wetting and drying rates, as well as the associated magnitudes of soil moisture response to NWS rainfall forecasts, the AEAR model can project out to time horizons of up to 24 h, perhaps longer, thus allowing for greater public awareness of potential landslide hazards.

### Future work

Although soil moisture is of critical importance across many fields, including agriculture, climate studies, landslide hazard assessments, landscape ecology, water resources, and wildfire management, widespread in situ soil moisture monitoring is somewhat recent. The challenge of analyzing and forecasting soil moisture presents an ongoing opportunity to apply recent advances in time series analysis and domain-driven data mining.

We plan to extend our methods to better incorporate lateral and vertical soil moisture variations. The timing and magnitude of response at different soil depths need to be examined over longer time periods incorporating multiple seasonal cycles to better constrain the AEAR parameters $k_s$ and $k_g$. Models should be applied to longer observation period data for investigation of large magnitude rainfall event response as well as to unburned field settings. As simple, physically based models cannot adequately represent the spectrum of physical properties in natural field settings, we will attempt to expand the model to forecast moisture over broader areas, creating a moisture map incorporating spatial variability of soil thickness and topographic position for potential application to agricultural water requirements, ecosystem response, burn susceptibility, and erosion response. By incorporating field and remotely sensed landscape data, as well as storm intensity and duration forecasts, future model improvements may aim to increase robustness of soil moisture and hence rainfall-induced landslide forecasts. Study of other evolutionary methods, including grammar-based methods, may prove beneficial in creating interpretable time-series data models [39].

Although our approach was designed for soil moisture forecasting, such models may be applied to other complex time-series applications in earth science and other areas. For example, managing workload bursts for network and computing resources are important to rebalancing and autoscaling for cloud computing [20, 70]. Workload bursts are a form of flash events (note similar language to “flash floods”) during major breaking news and sporting events of global interest, such as World Cup soccer [3, 6]. Web site traffic can fluctuate dramatically due to social network user activity [46]. Further, COVID-19 infection rates over time exhibit similarities with soil moisture. Time series of new confirmed cases [77] show comparable growth and decay functions as in Fig. 3. The SEIR (Susceptible–Exposed–Infected–Removed) epidemiological model, predicting epidemic COVID-19 trends, includes decay functions analogous to those of the NAR or AEAR models. Therefore, methods developed here including preprocessing time series observations, using accumulative models for forecasting, and interpreting physical processes using model parameters can potentially be applied to studying viral infections and aid in the design of appropriate control measures.

## Footnotes

1 http://landslides.usgs.gov/hazards/warningsys.php.

2 https://scihub.copernicus.eu/.

3 This article expands upon our prior conference publication [5] by providing: (i) comprehensive discussions of related work including a greater focus on explainability and interpretability; (ii) additional details on forecasting models and methods; (iii) specifics on the data analysis process in order to promote reproducibility; (iv) additional experimental results and discussion; (v) a broader experimental comparison with other machine learning, time-series, and soil moisture forecasting models including the long short-term memory (LSTM); and (vi) an expanded conclusion and discussion of future research.

4 Source code of novel models available at: https://github.com/olemengshoel/wetting-and-drying-of-soil.

5 https://www.wpc.ncep.noaa.gov/qpf/day1-7.shtml.

6 Data from USGS soil moisture probes installed in Winter Canyon on Pepperdine University, Malibu, CA. Location: CANVQSM-1 (UTM NAD83 Zone 11S, Easting Northing: 342754 3768790).

7 http://www.decagon.com/products/soils/volumetric-water-content-sensors/.

8 https://www.wunderground.com/history/.

9 https://cran.r-project.org/web/packages/RGA/index.html.

10 https://cran.r-project.org/web/packages/GenSA/index.html.

11 https://cran.r-project.org/web/packages/DEoptim/index.html.

12 https://cran.r-project.org/web/packages/irace/index.html.

13 https://stat.ethz.ch/R-manual/R-devel/library/stats/html/stl.html.

14 https://github.com/olemengshoel/hyperstl.
